# Cyclooxygenase-2 and Prostaglandin E_2_ Signaling through Prostaglandin Receptor EP-2 Favor the Development of Myocarditis during Acute *Trypanosoma cruzi* Infection

**DOI:** 10.1371/journal.pntd.0004025

**Published:** 2015-08-25

**Authors:** Néstor A. Guerrero, Mercedes Camacho, Luis Vila, Miguel A. Íñiguez, Carlos Chillón-Marinas, Henar Cuervo, Cristina Poveda, Manuel Fresno, Núria Gironès

**Affiliations:** 1 Centro de Biología Molecular Severo Ochoa, CSIC-UAM, Madrid, Spain; 2 Institut de Recerca de l'Hospital de la Santa Creu i de Sant Pau, Barcelona, Spain; 3 Instituto de Investigación Sanitaria de la Princesa, Madrid, Spain; 4 Department of Obstetrics/Gynecology, Columbia University Medical Center, Columbia University, New York, New York, United States of America; Albert Einstein College of Medicine, UNITED STATES

## Abstract

Inflammation plays an important role in the pathophysiology of Chagas disease, caused by *Trypanosoma cruzi*. Prostanoids are regulators of homeostasis and inflammation and are produced mainly by myeloid cells, being cyclooxygenases, COX-1 and COX-2, the key enzymes in their biosynthesis from arachidonic acid (AA). Here, we have investigated the expression of enzymes involved in AA metabolism during *T*. *cruzi* infection. Our results show an increase in the expression of several of these enzymes in acute *T*. *cruzi* infected heart. Interestingly, COX-2 was expressed by CD68^+^ myeloid heart-infiltrating cells. In addition, infiltrating myeloid CD11b^+^Ly6G^-^ cells purified from infected heart tissue express COX-2 and produce prostaglandin E_2_ (PGE_2_) *ex vivo*. *T*. *cruzi* infections in COX-2 or PGE_2_-dependent prostaglandin receptor EP-2 deficient mice indicate that both, COX-2 and EP-2 signaling contribute significantly to the heart leukocyte infiltration and to the release of chemokines and inflammatory cytokines in the heart of *T*. *cruzi* infected mice. In conclusion, COX-2 plays a detrimental role in acute Chagas disease myocarditis and points to COX-2 as a potential target for immune intervention.

## Author Summary

The role of prostanoids, products of the arachidonic acid pathway, during *Trypanosoma cruzi* infection has been studied by inhibiting key enzymes in prostanoid synthesis as cyclooxygenases (COX-1 and COX-2), with opposed results. Here we analyzed the expression of cyclooxygenases, prostanoid synthases and receptors in the heart of mice susceptible and non-susceptible to *T*. *cruzi* infection and found that they were highly increased respect to non-infected mice. We previously identified the presence of myeloid-derived suppressor cells expressing arginase-1 (Arg-1). Further analysis showed that COX-2 was expressed in Arg-1^-^ myeloid cells in heart tissue, suggesting the existence of different myeloid populations involved in the leukocyte infiltration (COX-2^+^Arg-1^-^) and tissue repair (COX-2^-^Arg-1^+^). Mice deficient in the expression of COX-2 and the prostaglandin PGE_2_ receptor EP-2 infected with *T*. *cruzi* showed a marked reduction in the cardiac inflammatory infiltration in comparison with infected wild type mice, indicating an adverse effect of COX-2 and PGE_2_ signaling through EP-2 receptor in the development of myocarditis during acute *T*. *cruzi* infection, suggesting the possibility of immune intervention using COX inhibitors.

## Introduction

Chagas disease is a multisystemic disorder caused by *Trypanosoma cruzi* infection that affects more than 8 million people worldwide, being endemic in Latin America. Due to the scarcity of preventive and therapeutic tools and population at risk, it is considered as a neglected tropical disease [1, 2]. More than 40,000 new infected people and 12,550 deaths per year are estimated. The high rate of migration towards non-endemic countries has spread the boundaries of the infection to other continents. Non-vectorial transmission is possible through oral ingestion, blood transfusion, organ transplantation and during pregnancy. The risk of infection is related to the country of origin of the migrants and the rate of prevalence in a given country [3].

Chagas disease is characterized by acute and chronic phases. Death occurs occasionally in the acute phase (<5–10% of symptomatic cases) as a result of severe myocarditis, meningoencephalitis, or both. The experimental model of infection in mice recapitulates many clinical features observed in human infection, although different strains of mice and parasites produce different disease outcomes [4].

Heart inflammation during the acute phase of *T*. *cruzi* experimental infection is initiated by lymphoid and myeloid mononuclear cell infiltration [5]. We have isolated from infected hearts an infiltrating monocytic CD11b^+^Ly6C^+^Ly6G^-^ population expressing both classical (M1) and alternatively (M2) activated macrophage markers that is able to suppress T cell proliferation *ex vivo*, characteristics that define them as myeloid-derived suppressor cells (MDSCs) [6, 7].

Myeloid cells are thought to be the major source of prostanoids, end products of cell membrane arachidonic acid (AA) catabolism, which include prostaglandins, prostacyclin and thromboxanes [8]. Enzymes implicated in prostanoid production have been investigated for many years [9]. All these lipid mediators have important roles in homeostasis and immune response regulation [10]. Cyclooxygenases, COX-1 and COX-2, are principal enzymes in prostanoid production. COX-1 expression is involved in homeostasis while COX-2 is induced by several factors, including infection [9]. However, the specific role of COX-2 and downstream enzymes in the context of infection varies depending on the infectious agent [11–13].

PGE_2_, a product of terminal PGE_2_ synthases (PGES) has pro-inflammatory properties [14] but also immunosuppressive properties [15] by signaling through G-protein coupled PGE receptors (EP), termed EP-1, EP-2, EP-3 and EP-4. PGE_2_ also decreases the ability of macrophages to phagocytize and kill microorganisms [16, 17], and is required for monocyte migration in response to chemokines [18, 19].

There are few studies about the role of prostanoids in human chagasic pathology [20, 21], but it has been described that monocyte inflammatory mediators inhibit cellular proliferation and enhance cytokine production in patients [22]. In rodent models of acute infection, the levels of PGF_2α_, TXB_2_, 6-oxo-PGF_1α_ [23] and PGE_2_ [24] in plasma, were increased. Macrophages from infected rats show an increased number of lipid bodies, where COX-2 produces PGE_2_ [25]. Recently, it has been shown that the absence of Phospholipase A2γ, an enzyme implicated in AA release from membranes, decreases mice survival [26].

The role of COX in mice infected with *T*. *cruzi* has been studied using non-selective inhibitors of COX-1 and COX-2, as well as COX-2-selective inhibitors (NSAIDs), with conflicting results. Thus, it has been described that COX inhibitors cause an increase in mortality and parasitism [27] in *T*. *cruzi* infection, but contrarily, other reports claim that COX-2 inhibition decreases the level of parasitism [28, 29]. In addition, both beneficial and adverse effects of COX inhibitors have been reported, depending on the phase of *T*. *cruzi* infection and the mice strain used [30]. Discrepancies between these studies could be explained by the different ability of BALB/c and C57BL/6 mouse strains to produce PGE_2_ [31]; the presence of distinct levels of cytokines in serum [32] or because of differences in cardiac cytokine expression profile [6].

Thus, in order to clarify the role of prostanoids in acute cardiac inflammation, we infected susceptible and non-susceptible mice, as well as COX-2 and EP-2 deficient mice with *T*. *cruzi* and analyzed cardiac inflammation, leukocyte infiltration and expression of cytokines, chemokines and inflammatory mediators in the infected mice.

## Results

### Prostanoid-synthesizing enzymes in the heart tissue of *T*. *cruzi* infected mice

**Fig 1:**
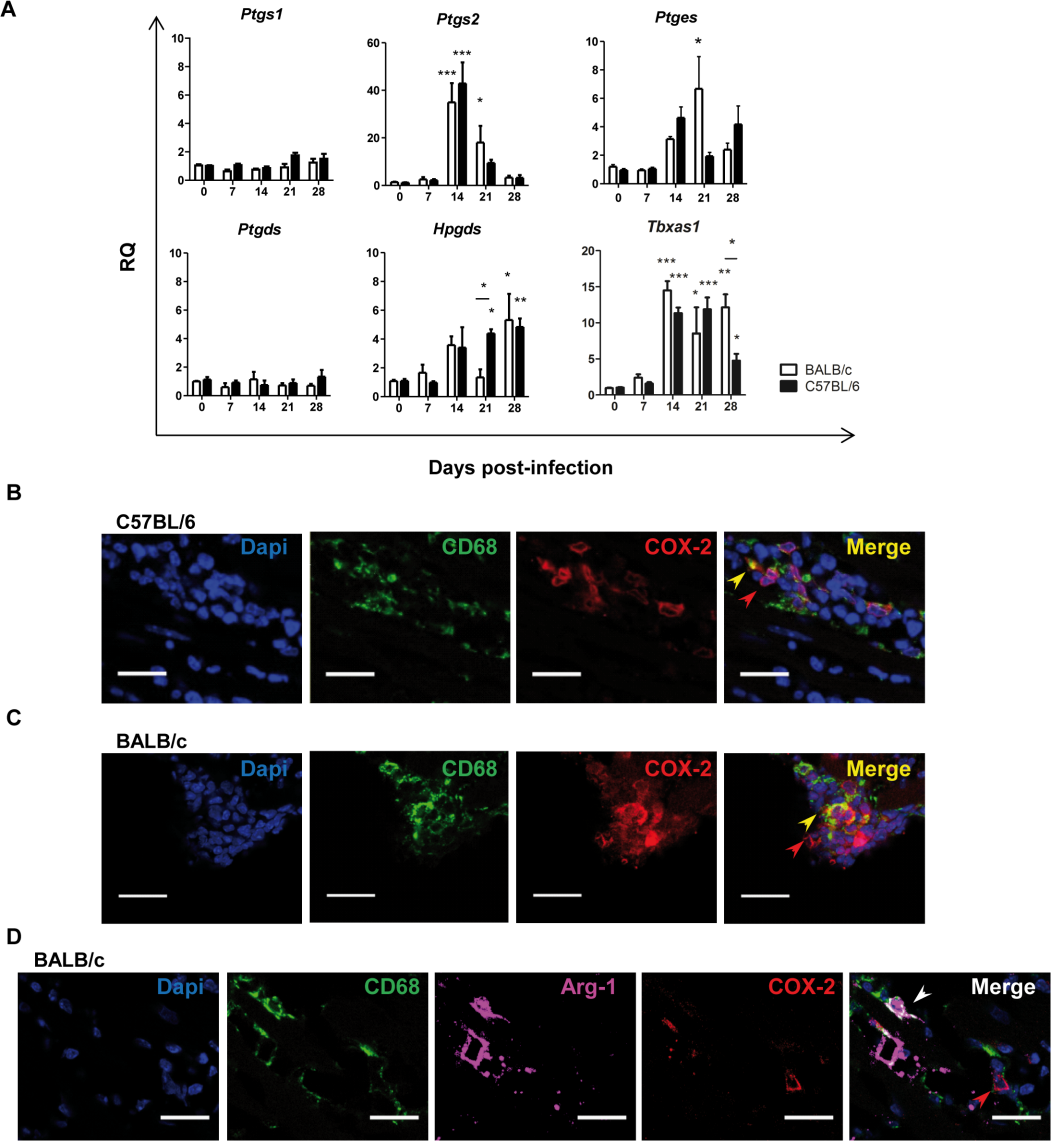
Expression of prostanoid synthases in *T*. *cruzi* infected cardiac tissue. (A) Expression of *Ptgs1*, *Ptgs2*, *Ptges* (mPGES1), *Ptgds*, *Hpgds* and *Tbxas1* in heart tissue of, either non-infected (0) or at different d.p.i. (7 to 28), BALB/c and C57BL/6 mice. Means ± SEM from three independent experiments (n = 9) are shown. Statistical comparisons are indicated * p< 0.05, *** p< 0.001. (B) Heart tissue samples from C57BL/6 mice at 14 d.p.i. and (C) BALB/c mice at 21 d.p.i., were stained with DAPI for nuclei (Blue) and specific antibodies for the macrophage marker CD68 (green) and the enzyme COX-2 (red). (D) Heart tissue from BALB/c mice at 21 d.p.i. stained for COX-2 (red), CD68 (green) and Arg-1 (magenta). In the merge of B, C and D, red, yellow and white arrows point to COX-2^+^, CD68^+^COX-2^+^ and CD68^+^Arg-1^+^ cells, respectively. Pictures are representative of several sections analyzed in 3 different mice from three independent experiments; the scale bar is 20 μm.

We infected mice with the Y strain of *T*. *cruzi*. Immunopathology caused by this parasite strain is characterized by cardiac inflammatory damage. As we previously reported [33], C57BL/6, but not BALB/c, infected mice recovered from infection and survived (S1A Fig). Parasitemia was detectable between 9 and 21 d.p.i. and was higher in BALB/c mice (S1B Fig). Hearts of BALB/c infected mice showed more leukocyte infiltration and parasite nests than C57BL/6 mice (S1C Fig). We next studied the expression level of enzymes involved in the AA pathway in the heart of both strains after infection (Fig 1A). COX-2 gene expression (*Ptgs2*), but not COX-1 (*Ptgs1*), was increased in heart tissue during the acute phase of *T*. *cruzi* infection similarly in both mouse strains. *Ptges* (microsomal prostaglandin E2 synthase, mPGES-1), *Hpgds* (leucocyte type PGD synthase) and *Tbxas1* (thromboxane synthase) mRNA expression levels were also incremented. However, *Ptgds* (lipocalin-type prostaglandin D synthase) mRNA basal level of expression in heart tissue did not change upon *T*. *cruzi* infection. These results indicate that *T*. *cruzi* infection promoted the selective up-regulation of some of the enzymes involved in prostanoid production in heart tissue, including COX-2 and mPGES-1.

### Myeloid cells in inflamed cardiac tissue express COX-2

Since COX-2 plays a key role in the synthesis of PGs in inflammatory processes, we aimed to identify the cells expressing this enzyme in the heart of *T cruzi* infected mice. Hearts from C57BL/6 (Fig 1B) and BALB/c (Fig 1C) mice were immunostained for COX-2 and myeloid and lymphoid markers, and imaged by confocal microscopy. Cells expressing COX-2 were abundant in the infected hearts of both mice strains, showing a strong staining in the perinuclear region of both myeloid CD68 positive and non-myeloid CD68 negative infiltrating cells. Interestingly, there was no co-localization of COX-2 and Arg-1, a marker of M2 macrophages and MDSCs (Fig 1D). Although COX-2 expression by activated lymphocytes has been previously described [34], CD4 staining was not detected in infiltrating COX-2^+^ cells in hearts of infected C57BL/6 (S2A Fig) nor BALB/c mice (S2C Fig). No staining was observed in negative control sections incubated with secondary antibodies alone (S2B, S2D and S2E Fig).

**Fig 2:**
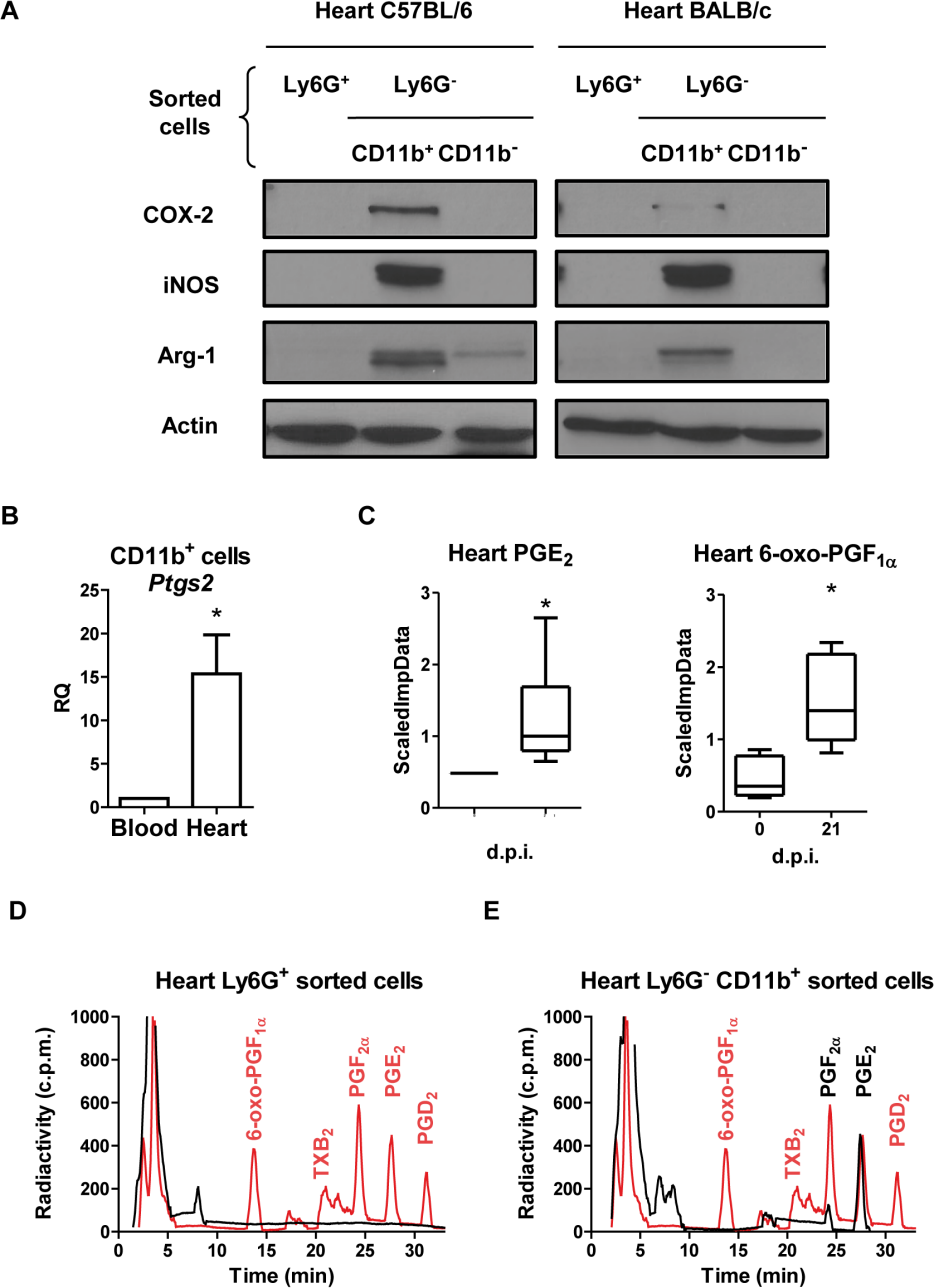
COX-2 expression and activity in heart and infiltrating myeloid cells during *T*. *cruzi* infection. (A) Purified Ly6G^+^, Ly6G^-^CD11b^+^and Ly6G^-^CD11b^-^ cells were obtained from infected C57BL/6 (14 d.p.i.) and BALB/c (21 d.p.i.) mice hearts, by magnetic cell separation. Arg-1, iNOS and COX-2 levels were analyzed by Western blot. Protein levels of Actin are shown as loading control. A representative experiment of the two performed is shown. (B) CD11b^+^ cells were obtained from pooled infected BALB/c mouse hearts or blood at 21 d.p.i (n = 15). *Ptgs2* (COX-2) expression in blood and heart tissue was determined by real time qRT-PCR. Mean ± SEM of two independent experiments is shown. (C) PGE_2_ and 6-oxo-PGF_1α_ levels in total heart extracts of BALB/c from non-infected (0 d.p.i.) and 21 d.p.i., were determined as described in Methods and represented as scaled imputed data (ScaledImpData) after normalizing raw data values respect to median values of each day run (*p<0.05). Prostanoid production in purified Ly6G^+^ (D) and Ly6G^-^ CD11b^+^ (E) cells from BALB/c mice hearts at 21 d.p.i. was determined by incubation with labeled 25 μM [C^14^] AA and analysis by HPLC (black line and text). Prostanoid standards (red line and text) were run in parallel as described in Methods. A representative experiment out of two performed is shown.

We next isolated myeloid cells from hearts of *T*. *cruzi* infected C57BL/6 and BALB/c mice at the times,14 and 21 d.p.i. respectively, when maximum Arg-1 and inducible nitric oxide synthase (iNOS) expression is observed [7]. Using anti-Ly6G antibody labeled magnetic microbeads we obtained the Ly6G^+^ population. CD11b^+^ cells were selected from the remaining Ly6G^-^ population, (Fig 2A). As previously described [7], the CD11b^+^Ly6G^-^ cell population expressed iNOS and Arg-1, and here we show that they also expressed COX-2 (Fig 2A). Interestingly, COX-2 gene expression was much higher in CD11b^+^ cells obtained from infected cardiac tissue than those from the blood (Fig 2B), pointing to infiltrating myeloid cells in inflamed tissue as the source of COX-2.

### CD11b^+^Ly6G^-^ cells from infected cardiac tissue produce PGE_2_

In agreement with the increase in COX-2 expression, a significant increase in the production of prostanoids as PGE_2_ and 6-oxo PGF1α in infected hearts was detected by mass spectrometry analysis on total heart extracts (Fig 2C). Further *ex vivo* analysis on Ly6G^+^ and CD11b^+^Ly6G^-^ purified heart infiltrating myeloid cells cultured in the presence of radiolabeled AA, showed that Ly6G^+^ cells did not produce any detectable prostanoid (Fig 2D). In contrast, CD11b^+^Ly6G^-^ cells produced high levels of PGE_2_ and low amounts of PGF_2α_ (Fig 2E). These results indicate that the CD11b^+^Ly6G^-^ myeloid population is able to synthesize high levels of PGE_2_ from AA, while other cell types in heart tissue are likely producing PGE_2_ and 6-oxo PGF1α.

### Cardiac inflammation is reduced in COX-2^-/-^ mice

**Fig 3:**
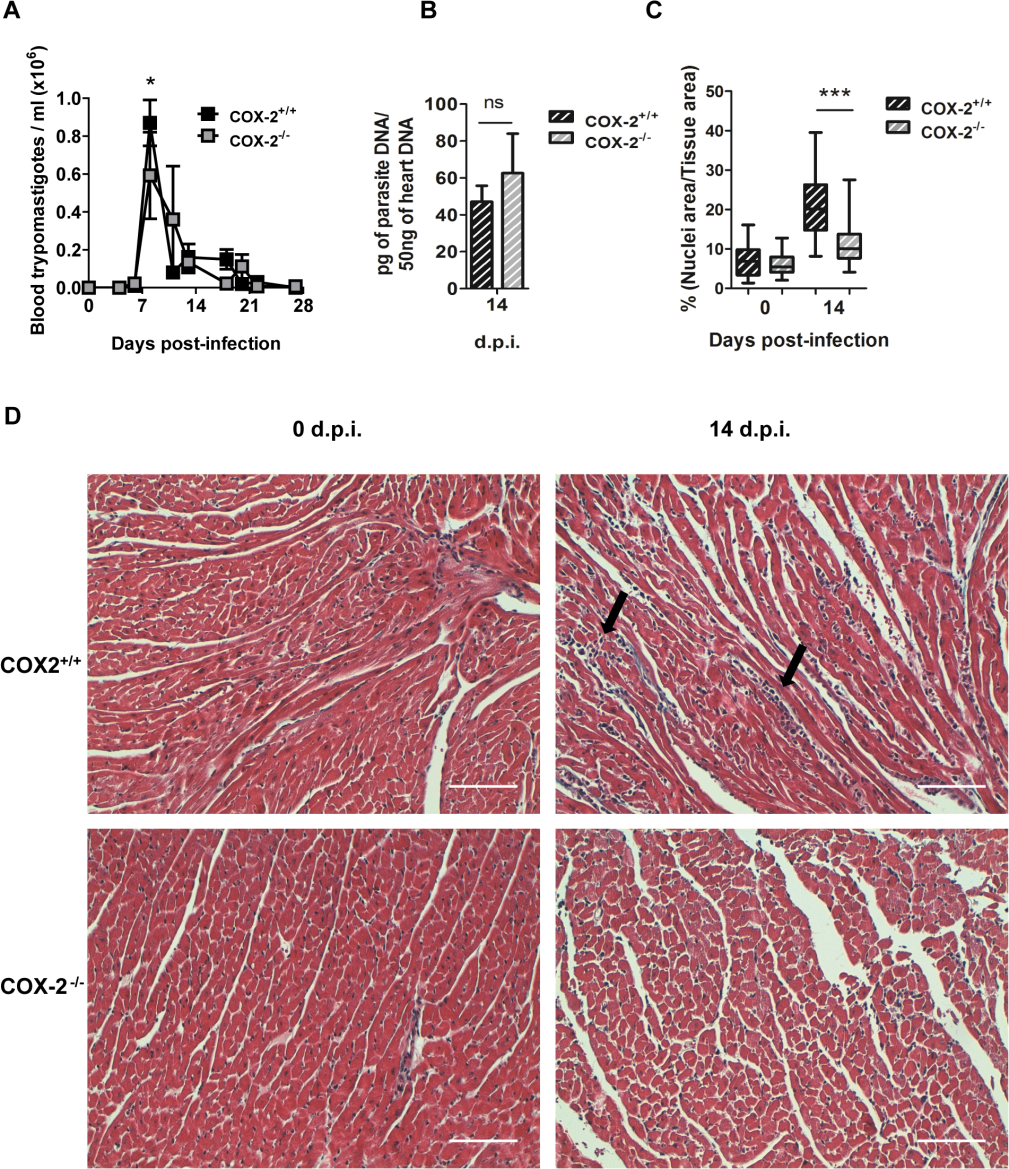
Parasite burden and heart inflammation in *T*. *cruzi* infected COX-2^+/+^ and COX-2^-/-^ mice. (A) The presence of parasites in the blood of COX-2^+/+^ or COX-2^-/-^ mice at different d.p.i. was quantified by direct counting under optical microscopy. (B) DNA from heart tissue was isolated and qPCR using *T*. *cruzi* DNA standard was performed to determine parasite burden in COX-2^+/+^ or COX-2^-/-^ infected mice at 14 d.p.i. Means ± SEM from three independent experiments are shown (n = 4). (C) Heart tissue sections of COX-2^+/+^ and COX-2^-/-^ mice either non-infected (0 d.p.i.) or 14 d.p.i., were stained with Masson’s Trichrome and inflammatory cell infiltration was quantified as described in Methods. (D) Representative pictures of heart tissue sections described in C. Arrows indicate inflammatory infiltration. Scale bar is 100 μm. (ns = non-significant; *p<0.05; **p< 0.01; ***p<0.001).

In order to study the role of COX-2 in the development of cardiac leukocyte infiltration, we infected COX-2^+/+^ and COX-2^-/-^ mice with the Y strain of the parasite. COX-2^-/-^ mice showed 30% reduction in blood parasite number compared to COX-2^+/+^ mice at the peak of parasitemia (Fig 3A). However, COX-2 deficiency did not significantly affect cardiac parasite burden compared to COX-2^+/+^ infected mice (Fig 3B). No mortality was observed neither in COX-2^+/+^ nor in COX-2^-/-^ infected mice up to 42 d.p.i.

Inflammatory infiltrates were analyzed and quantified in *T*. *cruzi* infected hearts from COX-2^+/+^ and COX-2^-/-^ mice. Fig 3C shows the extent of leukocyte infiltration calculated from several tissue sections as described in Methods. We observed significant less inflammatory infiltration in infected COX-2^-/-^ than in COX-2^+/+^ mice. Representative images corresponding to the quantification of cell infiltration are shown in Fig 3D.

**Fig 4:**
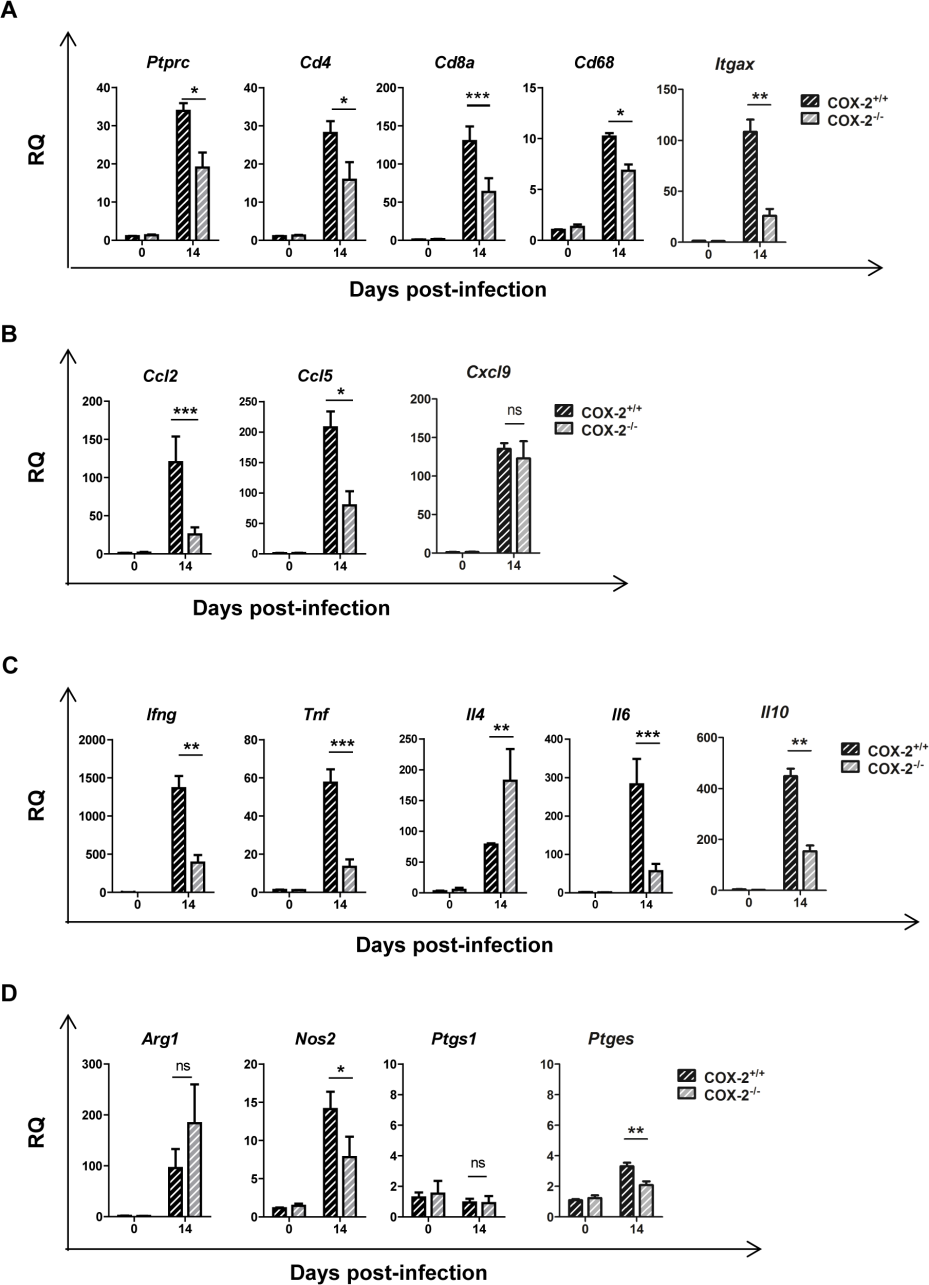
Gene expression of cell markers, chemokines, cytokines and inflammatory enzymes during *T*. *cruzi* infection in the heart of COX-2^+/+^ and COX-2^-/-^ mice. mRNA levels of the different genes analyzed was determined by qRT-PCR in heart tissue RNA samples isolated from non-infected (0 d.p.i.) or 14 d.p.i. COX-2^+/+^ or COX-2^-/-^ mice. Data are expressed as RQ calculated from CT values as described in Methods. Gene expression of lymphoid and myeloid cell markers as *Ptprc*, *Cd4*, *Cd8a*, *Cd68* and *Itgax* (A), chemokines as *Ccl2*, *Ccl5* and *Cxcl9* (B), cytokines as *Ifng*, *Tnf*, *Il4*, *Il6* and *Il10* (C) and enzymes as *Arg1*, *Nos2*, *Ptgs1* and *Ptges* (mPGES1) (D) is shown. Means ± SEM from one representative experiment (n = 3) out of four is shown (n = 5; * p<0.05; **p<0.01; ***p<0.001).

We next analyzed the cellular composition of the immune inflammatory infiltrate by determining gene expression of surface markers characteristic of various immune cell populations by qRT-PCR and normalizing the data from infected animals respect to non-infected controls. In agreement with histological findings, infection in COX-2^-/-^ mice compared to COX-2^+/+^ mice, led to lower expression of the common leukocyte marker *Ptprc* (CD45) as well as of *Cd4*, *Cd8*, *Cd68*, and *Itgax* (CD11c) as markers of T helper cells, cytotoxic T cells, macrophages and dendritic cells, respectively (Fig 4A).

**Fig 5:**
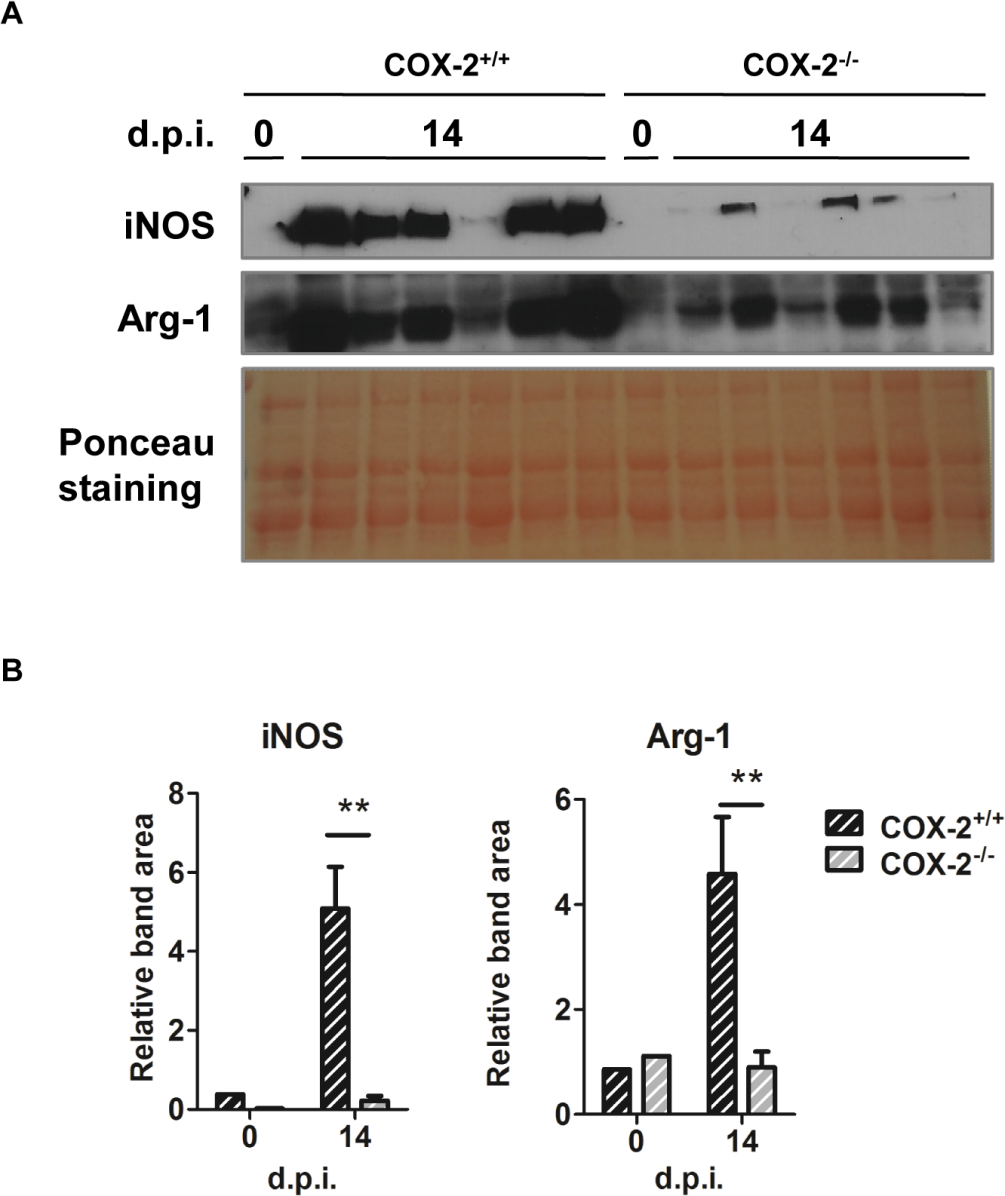
iNOS and Arg-1 expression in *T*. *cruzi* infected cardiac tissue of COX-2^+/+^ and COX-2^-/-^ mice. (A) Western blot analysis of iNOS and Arg-1 protein in extracts from hearts of COX-2^+/+^ and COX-2^-/-^ from non-infected mice (0 d.p.i.) and at 14 d.p.i. Ponceau staining of the blot is shown as a loading control. Samples for 6 different infected mice are shown. (B) Quantification of iNOS and Arg-1 band areas relative to the Ponceau staining from COX-2^+/+^ (dashed black bars) and COX-2^-/-^ (dashed gray bars) is represented as means ± SEM in arbitrary units. A representative experiment (n = 5) out of two is shown (**p<0.01).

To characterize the immune response in hearts of COX-2^-/-^ infected mice, gene expression of chemokines and cytokines were analyzed. mRNA levels of chemokines (*Ccl2*, *Ccl5* and *Cxcl9*) and cytokines (*Ifng*, *Tnf*, *Il4*, *Il6* and *Il10)* were significantly increased during *T*. *cruzi* infection in hearts of both COX-2^+/+^ and COX-2^-/-^ mice (Fig 4B and 4C). However, chemokine expression presented different patterns in COX-2^+/+^ and COX-2^-/-^ mice. *Ccl2* and *Ccl5* expression, but not *Cxcl9*, was significantly higher in COX-2^+/+^ mice than in COX-2^-/-^ mice (Fig 4B). Induction of pro-inflammatory cytokines *Ifng*, *Tnf* and *Il6* was lower, whereas *Il4* expression was higher, in COX-2^-/-^ compared to COX-2^+/+^ mice (Fig 4C). The anti-inflammatory cytokine *Il10* showed lower expression in the COX-2^-/-^ infected mice. There were no significant differences in *Arg1* expression between COX-2^+/+^ or COX-2^-/-^ mice (Fig 4D), but induction of *Nos2* mRNA (iNOS) was significantly lower in COX-2^-/-^ infected mice (Fig 4D). There was no induction of *Ptgs1* (COX-1) expression that could compensate for the COX-2^-/-^ deficiency (Fig 4D). *Ptges* (mPGES-1) expression was increased upon infection, with lower levels in heart tissue from COX-2^-/-^ mice compared to COX-2^+/+^ mice (Fig 4D). Nevertheless, protein analysis by western blot showed lower expression of both iNOS and Arg-1 in infected COX-2^-/-^ respect to COX-2^+/+^ mice (Fig 5). Analysis of TNF-α levels in plasma showed a similar increase in both COX-2^+/+^ and COX-2^-/-^ infected mice, indicating that the effect of COX-2 deficiency is not systemic but specific of the heart (S3A Fig). Basal levels of gene expression did not significantly change between COX-2^+/+^ and COX-2^-/-^ mice (S4 Fig).

### Cardiac inflammation is reduced in EP-2^-/-^ mice

**Fig 6:**
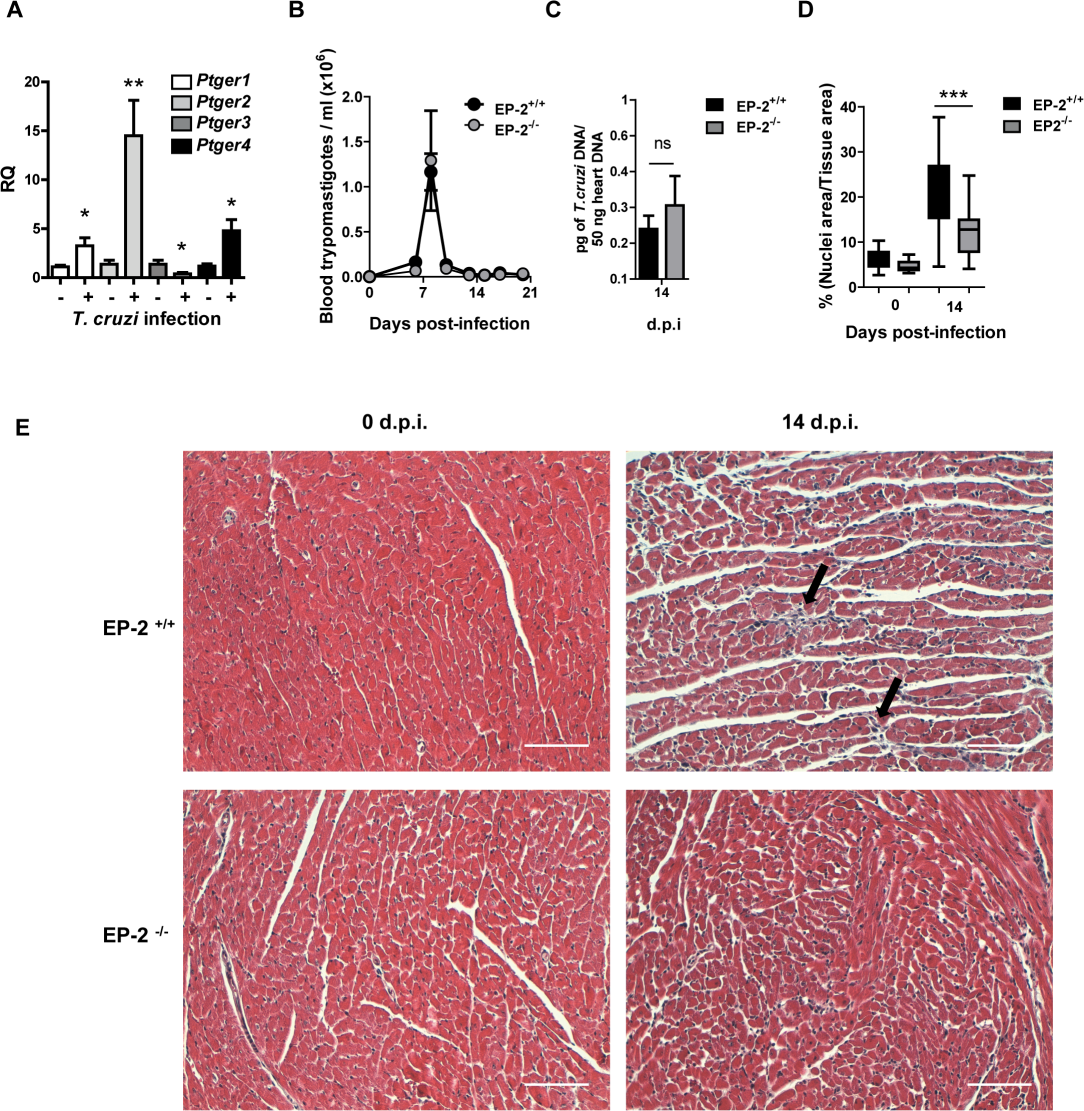
Parasite burden and heart inflammation in *T*. *cruzi* infected EP-2^+/+^ and EP-2^-/-^ mice. (A) mRNA levels of EP receptors (*Ptger1*, *Ptger2*, *Ptger3* and *Ptger4*) was determined by qRT-PCR in heart tissue RNA samples isolated from C57BL/6 mice non-infected (0 d.p.i.) and at 14 days post-infection (n = 5). (B) The presence of the parasites in the blood of EP-2^+/+^ or EP-2^-/-^ mice at different d.p.i. was quantified by direct counting under optical microscopy. (C) DNA from heart tissue was isolated and qPCR using *T*. *cruzi* DNA standard was performed to determine parasite burden in EP-2^+/+^ or EP-2^-/-^ infected mice at 14 d.p.i. Means ± SEM from a representative experiment (n = 4) from three independent experiments are shown (n = 4). (D) Heart tissue sections of EP-2^+/+^ and EP-2^-/-^ from non-infected mice (0 d.p.i.) and at 14 d.p.i., were stained with Masson’s Trichrome and inflammatory cell infiltration was quantified as described in Methods. (***p<0.001). (E) Representative pictures of heart sections described in C. Arrows indicate inflammatory infiltration. Scale bar is 100 μm.

We found increased levels of PGE_2_ in heart tissue and cardiac infiltrating cells after *T*. *cruzi* infection. Since the effector function of PGE_2_ produced by myeloid cells depends on its binding to EP receptors, we studied gene expression of its 4 receptors, EP-1 (*Ptger1*), EP-2 (*Ptger2*), EP-3 (*Ptger3*) and EP-4 (*Ptger4*), in hearts of mice during infection. The results show that in control infected mice the overall expression of EP receptors is higher than in non-infected hearts, except for *Ptger3* (Fig 6A). However, in C57BL/6 infected hearts *Ptger2* expression showed the highest increases suggesting a potential role of this receptor in *T*.*cruzi* infection. Thus, we used mice deficient in the expression of the EP-2 (in the C57BL/6 background), which has been involved in inflammation [35], and also in an autocrine loop of macrophage activation by PGE_2_ [36], in order to study the role of this receptor during *T*. *cruzi* infection. The results show that EP-2^+/+^ and EP-2^-/-^ mice survived infection and no significant differences in parasitemia or in heart parasite burden were observed between them (Fig 6B and 6C). These results suggest that EP-2 signaling does not play an essential role in parasite elimination.

**Fig 7:**
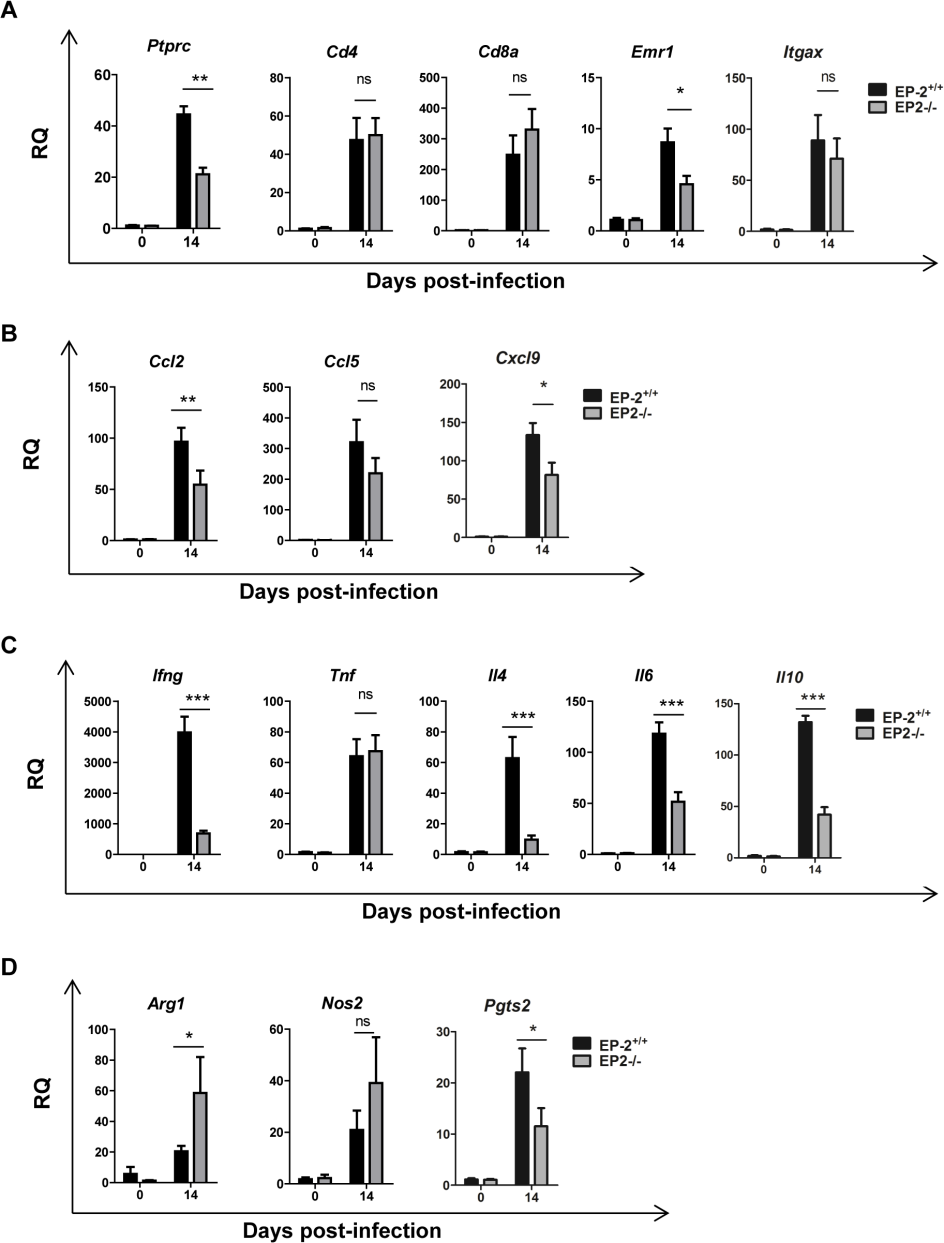
Gene expression of cell markers, chemokines, cytokines and inflammatory enzymes during *T*. *cruzi* infection in the heart of EP-2^+/+^ and EP-2^-/-^ mice. Expression of the different genes was analyzed by qRT-PCR in RNA samples of heart tissue isolated from EP-2^+/+^ and EP-2^-/-^ non-infected mice (0 d.p.i.) and at 14 d.p.i. Data are expressed as RQ calculated from CT values as described in Methods. Gene expression of lymphoid and myeloid cell markers as *Ptprc*, *Cd4*, *Cd8a*, *Cd68* and *Itgax* (A), chemokines as *Ccl2*, *Ccl5* and *Cxcl9* (B), cytokines as *Ifng*, *Tnf*, *Il4*, *Il6* and *Il10* (C) and enzymes as *Nos2*, *Ptgs2* and *Arg1* (D) is shown. Means ± SEM from a representative experiment (n = 3) out of two is shown (n = 4;* p<0.05; **p<0.01; ***p<0.001).

**Fig 8:**
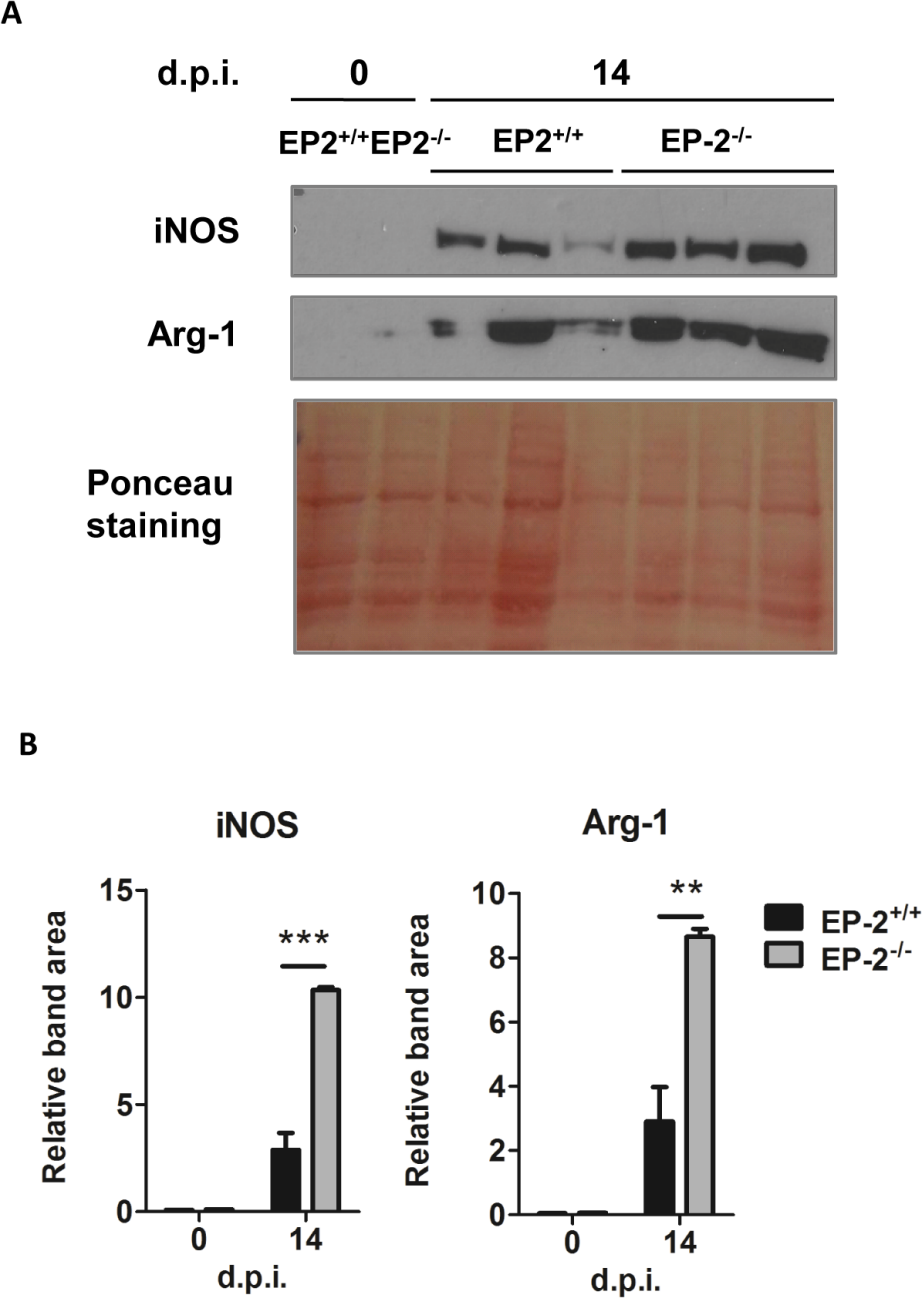
iNOS and Arg-1 expression in *T*. *cruzi* infected cardiac tissue of EP-2^+/+^ and EP-2^-/-^ mice. (A) Western blot analysis of iNOS and Arg-1 protein in extracts from hearts of EP-2^+/+^ and EP-2^-/-^ non-infected mice (0 d.p.i.) and at 14 d.p.i. Ponceau staining of the blot is shown as a loading control. Extracts from 3 different infected mice were loaded. (B) Quantification of iNOS and Arg-1 band areas relative to the Ponceau staining from EP-2^+/+^ (black bars) and EP-2^-/-^ (gray bars) is represented as means ± SEM in arbitrary units. A representative experiment out of two is shown (**p<0.01; ***p<0.001).

However, significant less heart inflammatory infiltrates were observed in infected EP-2^-/-^ in comparison with EP-2^+/+^ mice at 14 d.p.i. (Fig 6D). Representative images of cardiac tissue and inflammatory infiltration are shown in Fig 6E. The expression of the common leukocyte marker *Ptprc* (CD45) was lower in the heart of infected EP-2^-/-^ mice respect to EP-2^+/+^, whereas mRNA levels of cell markers as *Cd4* (Th cells), *Cd8* (Tc cells), and *Itgax*- (CD11c; DCs), did not show significant differences. However, the expression of *Cd68*, a macrophage marker, significantly increased in EP-2^-/-^ respect to EP-2^+/+^ mice (Fig 7A). Regarding chemokines, *Ccl2* expression, but not *Ccl5* and *Cxcl9*, was significantly reduced in the EP-2^-/-^ compared to EP-2^+/+^ infected mice (Fig 7B). Induction of pro-inflammatory cytokines *Ifng* and *Il6*, the Th2 cytokine *Il4* and the anti-inflammatory cytokine *Il10*, but not *Tnf*, was lower in EP-2^-/-^ compared with EP-2^+/+^ (Fig 7C). Similarly to COX-2^-/-^, no differences were observed in TNF-α plasma levels in EP-2^-/-^ as compared to EP-2^+/+^ infected mice (S3B Fig). *Ptgs2* (COX-2) gene expression was significantly lower in EP-2^-/-^ infected mice (Fig 7D). There were no differences between mouse strains in *Nos2* mRNA (iNOS) expression (Fig 7D), but *Arg1* mRNA expression was higher in EP-2^-/-^ mice (Fig 7D). Western blot analysis showed a significant increase in EP-2^-/-^ respect to EP-2^+/+^ mice, in the protein levels of these enzymes involved in L-arginine metabolism (Fig 8). Basal levels of gene expression did not significantly change between EP-2^+/+^ and EP-2^-/-^ mice (S5 Fig).

## Methods

### Parasites and mice

BALB/c and C57BL/6 mice (6 to 8-week-old) were purchased from Harlan, Interfauna Iberica. B6;129S7-*Ptgs2* ^*tm1Jed/J*^ (COX-2^-/-^) mice were purchased from The Jackson Laboratory. C57BL/6 *Ptger2* ^*tm1Sna*^ (EP-2^-/-^) mice were a gift form Dr. Shu Narumiya, (Faculty of Medicine, University of Kyoto). Wild type B6/129S (COX-2^+/+^) and C57BL/6 (EP-2^+/+^) mice were obtained by breeding heterozygote pairs.

*In vivo* infections were performed with Y *T*. *cruzi* strain as described [6, 7]. Groups of 3–15 mice were infected with 2,000 trypomastigotes per mice by intraperitoneal injection. Groups of 3–6 non-infected mice were included in the experiments as a control. Survival was monitored daily and parasitemia levels were checked every 2–3 days. Mice blood and tissues were collected at 0 (non-infected), 14 and 21 days post-infection (d.p.i.), as indicated.

### Ethics statement

This study was carried out in strict accordance with the European Commission legislation for the protection of animals used for scientific purposes (Directives 86/609/EEC and 2010/63/EU). Mice were maintained under pathogen-free conditions at the Centro de Biología Molecular Severo Ochoa (CSIC-UAM) animal facility. The protocol for the treatment of the animals was approved by the ‘‘Comité de Ética de Investigación de la Universidad Autónoma de Madrid”, Spain (permits CEI-14-283 and CEI-47-899). Animals had unlimited access to food and water. They were euthanized in a CO_2_ chamber and all efforts were made to minimize their suffering.

### Quantitative PCR

Hearts were perfused with Phosphate buffered saline (PBS) solution containing 1UI/ml of heparin, minced into small pieces with a sterile scalpel and DNA was isolated with High PurePCR Template preparation Kit (Roche). For *T*. *cruzi* detection, we used the quantitative PCR (qPCR) assay described by Piron et al. [37]. 100, 10, 1, 0.1 and 0.01 pg of DNA purified from Y strain epimastigotes were used to generate the standard curve. qPCR reactions were performed with 100 ng of genomic DNA and murine *Tnf* gene primers were used as DNA loading control.

### mRNA analysis by quantitative RT-PCR

For RNA extraction, heart tissue was perfused with PBS containing 1UI/ml of heparin, cut in small pieces using a sterile scalpel blade, followed by mechanical disruption using a PT 1300 D homogenizer (Kinematica Polytron, Fisher Scientific) in TRIzol reagent (Invitrogen) as indicated by the manufacturer. Gene expression was analyzed by quantitative reverse transcription PCR (qRT-PCR). Reverse transcription of total RNA was performed using the components of the High Capacity cDNA Archive Kit (Applied Biosystems. Life Sciences) or the SuperScript Enzyme (Invitrogen, Life Sciences). Amplification were performed using TaqMan MGB probes (S1 Table) and the TaqMan Universal PCR Master Mix (Applied Biosystems) on an ABI PRISM 7900 HT instrument (Applied Biosystems. Life Sciences). For cultured cells, samples were treated as mentioned above except for the mechanical disruption. All samples were assayed in triplicate. Quantification of gene expression by real-time PCR was calculated by the comparative threshold cycle (CT) method as described in [38] (RQ = 2^-ΔΔCT^). All quantifications were normalized to the ribosomal *18S* control to account for the variability in the initial concentration of RNA and in the conversion efficiency of the reverse transcription reaction.

### Protein expression analysis

Protein extracts were prepared from heart tissue perfused with PBS containing 1UI/ml of heparin, cut in small pieces using a sterile scalpel blade followed by mechanical disruption in Triton X-100 based protein lysis buffer. Protein concentration was determined by the bicinchoninic acid method (BCA, Pierce) using Bovine Serum Albumin (BSA) for the standard curve. Western blot analyses were performed as follows: 15 or 50 μg of cell or tissue extract were fractionated on SDS polyacrylamide gel and transferred to a Nitrocellulose membrane Hybond-ECL (Amersham Biosciences) and blocked in 5% fat free dry milk or 5% BSA in 0,1% Tween-20 Tris Buffered Saline. Membranes were incubated overnight with diluted primary antibodies (S2 Table) at 4–8°C. The membranes were incubated with horseradish peroxidase conjugated secondary antibodies (S2 Table) and detection was carried out with Supersignal detection reagent (Pierce) followed by photographic film exposure. Fiji package software was used to quantify band intensity normalizing band areas of the sample to their respective loading control.

### Histological studies

Cardiac tissues from mice were placed in 10% neutral buffered formalin for at least 4 hours at room temperature followed by incubation in 70% ethanol overnight. Samples were then embedded in paraffin (Tissue Embedding Station Leica EG1160), and 5 μm tissue sections were prepared (Microtome Leica RM2155). Samples were deparaffinized and rehydrated using a Tissue Processing Station Leica TP1020. Slides were stained with Masson´s Trichrome staining and mounted permanently in Eukitt´s quick hardening mounting medium (Biochemika, Fluka analytical). The sections were microscopically analyzed in a Leica microscope (DMD 108, Leica microsystems Wetzlar GmbH, Germany) using the 20x magnification objective lens and Lamp intensity 10 and f/Stop 12. Ventricular myocardium micrographs were taken avoiding pericardium, endocardium, atria and big vessels. Nine pictures of different sections, separated by at least 50 μm, per heart were taken. The degree of inflammatory-cell infiltration was quantified using the Fiji package [39] and the plugin Trainable Weka Segmentation developed by Ignacio Arganda Carreras (Versailles, France) [39] (Image J macro used for automated image analysis is detailed in S1 File) and expressed as the percentage of the nuclei/tissue area ratio.

### Confocal immunofluorescence

Organs were removed from mice at different d.p.i., cut and fixed in a 4% paraformaldehyde PBS buffered solution for 2h at room temperature, followed by incubation in a 30% sucrose solution at 4°C overnight. Tissues were then embedded in Tissue-Tek OCT in Cryomolds (Sakura) and frozen. 10 μm sections were cut using a cryostat Leica CM1900. Slides were fixed in acetone for 10 min at room temperature and incubated 10 min with NH_4_Cl to reduce autofluorescence. Then, slides were washed with PBS, permeabilized with 0,1% Triton X-100, blocked and incubated over night at 4°C with primary antibodies (S2 Table) in blocking buffer (PBS 0,1% Triton X-100, 5% BSA). The samples were washed with PBS and secondary antibodies (S2 Table) were added in blocking buffer and incubated overnight at 4°C. Blocking of unspecific secondary antibody binding was achieved by addition of 2% of normal serum of the species in which the secondary antibody was raised. As a negative control, sections were treated in the same manner, except that incubation with primary antibody was omitted. Nuclei were stained using 1 μg/ml of DAPI (268298, Merck). Prolong Gold Antifade Reagent (Invitrogen) was used to mount the slides that were kept at 4°C until observation. Stained slides were observed with the confocal laser scanning microscope LSM710, coupled to an AxioimagerM2 microscope (Zeiss). The micrographs were processed using the software ZEN (Zeiss) or the Fiji Package.

### Isolation of Ly6G^+^ and CD11b^+^Ly6G^-^ cells by magnetic sorting

BALB/c (n = 15) or C57BL/6 mice (n = 15) were infected i.p. with 2,000 trypomastigotes of the Y strain. At 21 d.p.i. for BALB/c and 14 d.p.i. for C57BL/6, mice were euthanized in a CO_2_ chamber and hearts were aseptically removed, perfused with 10 ml PBS containing 1UI/ml of heparin, and kept in cold Hank´s balanced saline solution (HBSS). Then, hearts were pooled in a cell culture dish, washed thoroughly with HBSS and minced into small pieces with a sterile surgery blade. Mouse hearts (maximum 4 per tube) were transferred into the gentleMACS C tube containing 4,7 mL of HBSS. 300μL of Collagenase II solution (600 U/ml) and 10 μl DNase I solution (60U/ml) were added. Then tissue was disrupted with GentleMACS Dissociator (Miltenyi Biotec GMbH). To obtain cell suspensions, a 70 μm cell strainer (Falcon BD) was used. After red blood cells lysis, the cells were magnetically sorted. For Ly6G^+^ cell sorting, anti Ly-6G MicroBead kit was used with MACS LS columns and MACS Separators (Miltenyi Biotec GmbH), following manufacturer instructions. Ly6G^-^ fraction of the cell suspension was afterwards processed for CD11b^+^ cell sorting using CD11b Microbeads kit.

### Analysis of prostanoids

Prostanoid levels were determined in mouse tissue extracts from 0 (non-infected) and 21 d.p.i. by Metabolon Inc., and expressed as ScaledImpData as previously described [40]. To determine *in vitro* prostanoid production, heart infiltrating cells were magnetically sorted as described above and incubated 30 minutes at 37°C in 500 μl of RPMI without Fetal bovine serum (FBS) in the presence of 25 μM [^14^C] AA, PerkinElmer (Massachusetts, USA). 500 μl of 2% acetic acid in cold methanol was added to extract and preserve AA derivatives. Samples were vortexed and the air inside the tube was substituted by inert nitrogen gas. Samples were kept frozen at -80°C until HPLC was performed. HPLC device was composed by a Beckman Solvent Module 126 with the column Ultraphere ODS (C-18, Beckman-Coulter) 5 μm diameter sphere particle, 4.5 mm and 25 cm column diameter and length respectively and a Beckman 171 Radioisotope Detector. Scintillation liquid Ecoscint H was purchased from National Diagnostics. Prostanoids were resolved with the isocratic flow (1ml/min) of the mobile phase: Acetonitrile/water/acetic acid 33:67:0.1 v/v/v. Standards were produced using [^14^C] Arachidonic Acid and different cell types expressing the respective enzymes, and [^14^C] Arachidonic Acid incubated in medium was used as input control as described [41].

### Statistical analysis

For *in vivo* experiments, data are shown as means ± SEM. All the *in vitro* experiments were performed at least three times. Significance was evaluated by Student’s t-test when two groups were compared. ANOVA one way followed by Tukey post-test was used when groups of samples from an experiment had different time points. ANOVA two way followed by Bonferroni post-test were used when the experiment included time and mice strain as variables. For survival analysis, we used Gehan-Breslow-Wilcoxon method. GraphPad Prism 5.00 software (La Jolla, CA, USA) was used for statistical analysis.

## Discussion

In order to clarify the role of prostanoids in the outcome of T. cruzi infection we first analyzed the expression of prostanoid-synthesizing enzymes in cardiac tissue from T. cruzi susceptible (BALB/c) and non-susceptible (C57BL/6) mice. Our results showed an increase of COX-2/mPGES-1/PGE_2_ axis in heart tissue upon infection in both strains of mice, indicating that it has no direct effect on susceptibility to infection. Confocal microscopy analysis showed the presence of CD68^+^Arg-1^+^COX-2^-^ cells and CD68^+^Arg-1^-^COX-2^+^, suggesting that there are at least two subpopulations of monocytic infiltrating cells with mutually exclusive expression of those enzymes. Thus, our results show that the myeloid population infiltrating the heart in *T*. *cruzi* infection is more complex than previously described [7], and suggests a difference in the function of these two myeloid populations. Macrophages can rapidly change their phenotype and function in response to local microenvironmental signals, playing key roles in the initiation and resolution of inflammation and tissue homeostasis [42] and could be involved in tissue repair and fibrosis [43]. Thus, myeloid cardiac infiltration could inhibit parasite replication and also facilitate the repair of damaged muscular tissue [44]. A suggestive hypothesis is that COX-2 expressing macrophages could be linked to inflammation meanwhile Arg-1^+^ macrophages could be involved in tissue repair.

Immunostaining of heart tissue sections showed the presence of myeloid and non-myeloid cells positive for COX-2 in heart tissue sections of both BALB/c and C57BL/6 mice. However, after purification of myeloid cells from heart tissue, we found that only a particular subset expressed COX-2, being the levels of COX-2 expression higher in C57BL/6 than BALB/c mice. Likely, non-myeloid COX-2 positive cells are lost in the purification process, a fact that might account for the apparent contrary results.

Interestingly, we demonstrated that PGE_2_ and 6-oxo-PGF_1α_ (stable hydrolysis product of PGI_2_) were elevated in infected heart tissue. Furthermore, monocytes (CD11b^+^Ly6G^-^) isolated from infected heart express COX-2, and are able to produce high levels of PGE_2_ *ex vivo*. The differences in metabolites detected in total heart extracts *versus* purified myeloid cells are likely due to their synthesis by other cell types and/or enrichment after cell purification. In agreement with this, we have previously reported that *T*. *cruzi* infection induces COX-2 in cardiomyocytes, leading to PGF_2α_ and TXA_2_ production [45]. Although COX-2 expression can be induced in CD4^+^ T cells upon activation [34], heart infiltrating CD4^+^ cells did not express detectable levels of COX-2.

On the other hand, the increase of *Tbxas1* and *Hpgds* gene expression observed upon *T*. *cruzi* infection in both C57BL/6 and BALB/c mice suggests the production of their respective TXA_2_ and PGD_2_ metabolites in infected cardiac tissue. *Tbxas1* was elevated up to 28 d.p.i. and its product, TXA_2_, besides its vascular functions [46], could have a pro-inflammatory role for monocytes [47]. In contrast, *Hpgds* expression showed a gradual increase during the acute phase, and its product, PGD_2_, could be involved in resolution of inflammation, as described in other settings [48–50]. Resistant C57BL/6 mice showed significantly higher expression of *Hpgds* at 21 d.p.i. and lower expression of *Tbxas1* at 28 d.p.i. than the susceptible BALB/c mice, suggesting that C57BL/6 may resolve inflammation earlier than BALB/c infected mice. However, TXA_2_ and PGD_2_ metabolites were not detected in purified Ly6G^+^ nor in CD11b^+^Ly6G^-^ cells, suggesting that they could be produced by other infiltrating cell types or by infected cardiomyocytes [45].

Previous reports using COX-2 inhibitors in *T*. *cruzi* infection showed discordant results [30]. Moreover, COX-2 inhibitors may interfere with the immune response [34], but more importantly, many COX-2 inhibitors have effects independent of their ability to inhibit cyclooxygenase activity [51–53]. For those reasons, we analyzed the contribution of COX-2 by using a mouse model deficient for its expression. We found a small variation in parasitemia (30% reduction at the peak of parasitemia) in COX-2^-/-^ respect to the COX-2^+/+^ mice, which cannot be taken as indicative of resistance. In addition, no changes were observed in cardiac parasite burden, in spite that COX-2^-/-^ mice expressed less iNOS than COX-2^+/+^ mice, considered to be key for resistance in *T*. *cruzi* infection [54], indicating that heart parasite load is not affected by the lack of COX-2 expression. Interestingly, we found that COX-2 was required for leukocyte infiltration and inflammation in the heart upon *T*. *cruzi* infection, but it did not affect systemic inflammation. However, COX-2^-/-^ mice are resistant to death in sepsis, indicating that in this case COX-2 has a systemic pro-inflammatory role [55]. An important role of endogenous COX-2 derived PGs in migration of immune cells to infected tissues or lymphoid organs is becoming evident [19]. Thus, the decrease in cardiac inflammation and in local production of cytokines and chemokines observed in COX-2^-/-^ infected mice, indicate a pro-inflammatory role of prostanoids as PGE_2_ in acute myocarditis.

We have previously described that PGE_2_ induces COX-2 and mPGES-1 expression in an autocrine loop required for full activation of macrophages [36]. Thus, the fact that COX-2^-/-^ mice showed a reduced *Ptges* mRNA expression (mPGES-1) suggest a blockade of the autocrine loop that may impair full activation of macrophages, resulting in the reduced cardiac infiltration observed. In the same direction, lack of PGE_2_ signaling through EP-2 receptor in EP-2^-/-^ infected mice, resulted in reduced *Ptgs* (COX-2) mRNA expression that may also block the autocrine loop, impairing macrophages to infiltrate the cardiac tissue. This is in agreement with the observed decrease in *Ccl2* mRNA expression in COX-2^-/-^ and EP-2^-/-^ infected mice respect to wild type infected mice, since this chemokine is required for migration of monocytes to the inflamed infected tissue [56]. Moreover, PGE_2_ also affects migration of myeloid cells potentiating CCL2 activity [19]. Therefore, the reduction of cardiac infiltration in both animal models suggest a detrimental pro-inflammatory role of COX-2 in the onset of cardiac inflammation.

Strikingly, Arg-1 and iNOS expression, markers of MDSCs, was higher in hearts of infected EP-2^-/-^ mice than in those from COX-2^-/-^ infected mice hearts, indicating that infiltrating cells from EP-2^-/-^ mice present a more marked MDSCs phenotype. In addition, the effect of EP-2 deficiency on cytokine and chemokine production in heart, was milder than the observed in COX-2^-/-^ infected mice. In the hearts of C57BL/6 mice, infection caused a greater significant increase in the expression of *Ptger2*, which validates the use of EP-2^-/-^ mice in the C57BL/6 background. But still *Ptger1* and *Ptger4* were significantly increased although in a minor extent. Thus in EP-2^-/-^ infected mice PGE_2_ can still signal through *Ptger1* and *Ptger4* causing this milder effect in EP-2^-/-^ mice in comparison to the observed in COX-2^-/-^ infected hearts. In contrast, in COX-2^-/-^ infected mice, mPGES1 synthase expression is substantially reduced and PGH_2_ substrate for PGE_2_ production likely relies on constitutive COX-1 activity. Thus, the decreased levels of PGE_2_ may affect signaling through all PGE_2_ receptors, having a broader effect on leukocyte infiltration. The response to *T*. *cruzi* infection in mice deficient in other enzymes and products of the AA pathway has been scarcely studied [26, 27]. Sharma *et al*. described that deficiency of iPLA_2-_γ (Ca^++^ independent PLA_2_ isoform-γ), which is involved in AA membrane release, aggravated infection and decreased survival, while Mukherjee *et al*. described that COX-1^-/-^ mice showed higher parasitemia than wild type infected mice, but no difference in survival was noted [27]. In our hands, interference within the AA pathway at a different level, as COX-2 mediated production of prostanoids or PGE_2_/EP-2 signaling, results in decreased inflammation in heart of *T*. *cruzi* infected mice, with low incidence in parasite burden and survival. Altogether, these results point to an essential role of the AA pathway in heart inflammation during *T*. *cruzi* infection.

In the other hand, related with the prostanoid pathway, 5-lipoxygenase (5-LO) has been shown to play a detrimental role during T. cruzi infection by potentiating heart parasitism and inflammation [57, 58] through the regulation of iNOS activity [59]. However, further studies are needed to elucidate the crosstalk between LO and COX pathways during infection.”

Besides, we have previously showed that monocytic CD11b^+^Ly6G^-^ heart infiltrating cells (MDSCs), expressing iNOS and Arg-1 suppressed *ex vivo* T cell proliferation [7]. Since some of these infiltrating cells also express COX-2 and produce PGE_2_ it is possible that COX-2-derived PGs could contribute to immune suppression, a possibility that should be addressed in the future.

In conclusion, during acute *T*. *cruzi* infection there is an increase in the expression of many enzymes of the AA metabolism, including COX-2 and mPGES-1 that leads to an increase in their metabolite PGE_2_, partially due to infiltrating myeloid cells in the heart. Besides, we have identified a new myeloid infiltrating population characterized by the expression of COX-2. Thus, so far there are at least three different myeloid populations infiltrating the *T*. *cruzi* infected heart: granulocytes, monocytic MDSCs expressing iNOS and Arg-1 and monocytic cells expressing COX-2. COX-2 activity likely increases PGE_2_ levels in heart tissue, which play a pro-inflammatory role by signaling through EP-2. However, the phenotype of EP-2^-/-^ is not as strong as COX-2^-/-^ infected mice probably due to PGE_2_ signaling through alternative EP receptors. Our findings suggest that COX-2 plays a detrimental role in acute Chagas disease myocarditis. Further research of the AA pathway is needed to completely understand its role during *T*. *cruzi* infection for immune intervention approaches.

## Supporting Information

S1 Fig Survival, parasite burden and inflammation of BALB/c and C57BL/6 mice infected with *T*. *cruzi*. (A) Survival was checked every day during the infection in BALB/c and C57BL/6 mice (n = 5). (B) The presence of the parasites in the blood of BALB/c and C57BL/6 mice was quantified by direct counting under optical microscopy. Means ± SEM of a representative experiment (n = 4) from two independent experiments are shown. (* p< 0.05; ** p< 0.01). (C) Histology of cardiac tissue in non-infected (0 d.p.i.) or 21 d.p.i *T*. *cruzi* infected BALB/c and C57BL/6 mice. Representative pictures of heart tissue sections of each group, processed for Masson`s Trichrome histology staining, are shown. Black arrows point to the infiltrating leukocytes and red arrows point to parasite nests. Scale bar is 100 μm.
(TIF)

S2 Fig COX-2 and CD4 expression in *T*. *cruzi* infected mouse cardiac tissue. (A) Heart tissue was isolated at 14 d.p.i. from infected C57BL/6 mice and sections were stained with DAPI for nuclei (Blue), the lymphocyte marker CD4 (green) or COX-2 (red). A representative picture of several sections analyzed in at least three different mice from two independent experiments is shown. (B) Control sample from heart of C57BL/6 mice at 14 d.p.i. incubated with secondary antibodies coupled to Alexa Fluor (AF) 488, and 647 in the absence of primary antibodies. (C) Same as in A from heart of BALB/c mice at 21 d.p.i.. (D) Same as in B from heart of BALB/c mice at 21 d.p.i. (E) Control samples from heart of BALB/c mice at 21 d.p.i. incubated with secondary antibodies coupled to Alexa Fluor (AF) 488, 555 and 647 in the absence of primary antibodies. Scale bar is 20 μm.
(TIF)

S3 Fig Serum levels of TNFα in COX-2^+/+^, COX-2^-/-^, EP-2^+/+^ and EP-2^-/-^ mice during *T*. *cruzi* infection. TNFα concentration in non-infected mice (0 d.p.i.) and at 14 d.p.i. in blood serum of (A) COX-2^+/+^ and COX-2^-/-^ and (B) EP-2^+/+^ and EP-2^-/-^ mice. Representative means ± SEM from two independent experiments are shown (n = 6) (ns = non-significant).
(TIF)

S4 Fig Comparison of the gene expression of cell markers, chemokines, cytokines and inflammatory enzymes basal levels in the heart of COX-2^+/+^ and COX-2^-/-^ mice. mRNA levels of the different genes analyzed was determined by qRT-PCR in heart tissue RNA samples isolated from non-infected (0 d.p.i.) COX-2^+/+^ or COX-2^-/-^ mice. Data are expressed as RQ calculated from CT values as described in Methods. Gene expression of lymphoid and myeloid cell markers as *Ptprc*, *Cd4*, *Cd8a*, *Cd68* and *Itgax* (A), chemokines as *Ccl2*, *Ccl5* and *Cxcl9* (B), cytokines as *Ifng*, *Tnf*, *Il4*, *Il6* and *Il10* (C) and enzymes as *Arg1*, *Nos2*, *Ptgs1* and *Ptges* (mPGES1) (D) is shown. Means ± SEM from one representative experiment (n = 3) out of four is shown (n = 5; * p<0.05).
(TIF)

S5 Fig Comparison of the gene expression of cell markers, chemokines, cytokines and inflammatory enzymes basal levels in the heart of EP-2^+/+^ and EP-2^-/-^ mice. mRNA levels of the different genes analyzed was determined by qRT-PCR in heart tissue RNA samples isolated from non-infected (0 d.p.i.) EP-2^+/+^ or EP-2^-/-^ mice. Data are expressed as RQ calculated from CT values as described in Methods. Gene expression of lymphoid and myeloid cell markers as *Ptprc*, *Cd4*, *Cd8a*, *Cd68* and *Itgax* (A), chemokines as *Ccl2*, *Ccl5* and *Cxcl9* (B), cytokines as *Ifng*, *Tnf*, *Il4*, *Il6* and *Il10* (C) and enzymes as *Nos2*, *Ptgs2* and *Arg1* (D) is shown. Means ± SEM from one representative experiment (n = 3) out of four is shown (n = 5; * p<0.05).
(TIF)

S1 Table Taqman probes. List of the Taqman Probes from Applied Biosystems (A&B) used in the mRNA analysis by quantitative RT-PCR, including reference of the manufacturer, gene symbol and protein name.
(PDF)

S2 Table Antibodies. List of antibodies from different providers used in confocal immunofluorescence and western blot analysis including the reference from each provider, the application and the dilution utilized.
(PDF)

S1 File Image J macro used for automated image analysis. Plugin Trainable Weka Segmentation developed by Ignacio Arganda Carreras (Versailles, France).
(PDF)
