# Correction: Cyclooxygenase-2 and Prostaglandin E2 Signaling through Prostaglandin Receptor EP-2 Favor the Development of Myocarditis during Acute *Trypanosoma cruzi* Infection

**DOI:** 10.1371/journal.pntd.0004175

**Published:** 2015-10-20

**Authors:** Néstor A. Guerrero, Mercedes Camacho, Luis Vila, Miguel A. Íñiguez, Carlos Chillón-Marinas, Henar Cuervo, Cristina Poveda, Manuel Fresno, Núria Gironès

The second sentence in the seventh paragraph of the Introduction should have cited references 27 and 28 in the first part of the sentence, and should have cited reference 29 at the end of the sentence. The correct sentence should read: Thus, it has been described that COX inhibitors cause an increase in mortality and parasitism [27, 28] in T. cruzi infection, but contrarily, other reports claim that COX-2 inhibition decreases the level of parasitism [29].

According to this new order, references 28 and 29 should appear in the References as follows:

28. Hideko Tatakihara VL, Cecchini R, Borges CL, Malvezi AD, Graca-de Souza VK, Yamada-Ogatta SF,et al. Effects of cyclooxygenase inhibitors on parasite burden, anemia and oxidative stress in murine Trypanosoma cruzi infection. FEMS immunology and medical microbiology. 2008; 52(1):47–58. PMID:_18031539_.

29. Freire-de-Lima CG, Nascimento DO, Soares MB, Bozza PT, Castro-Faria-Neto HC, de Mello FG, et al.Uptake of apoptotic cells drives the growth of a pathogenic trypanosome in macrophages. Nature.2000; 403(6766):199–203. PMID: _10646605_.
