# Supplementary figures and images for: Cyclooxygenase-2 and Prostaglandin E2 Signaling through Prostaglandin Receptor EP-2 Favor the Development of Myocarditis during Acute Trypanosoma cruzi Infection
Source: PLoS Negl Trop Dis. 2015 Aug 25;9(8):e0004025. doi: 10.1371/journal.pntd.0004025 (PMC4549243; doi:10.1371/journal.pntd.0004025)

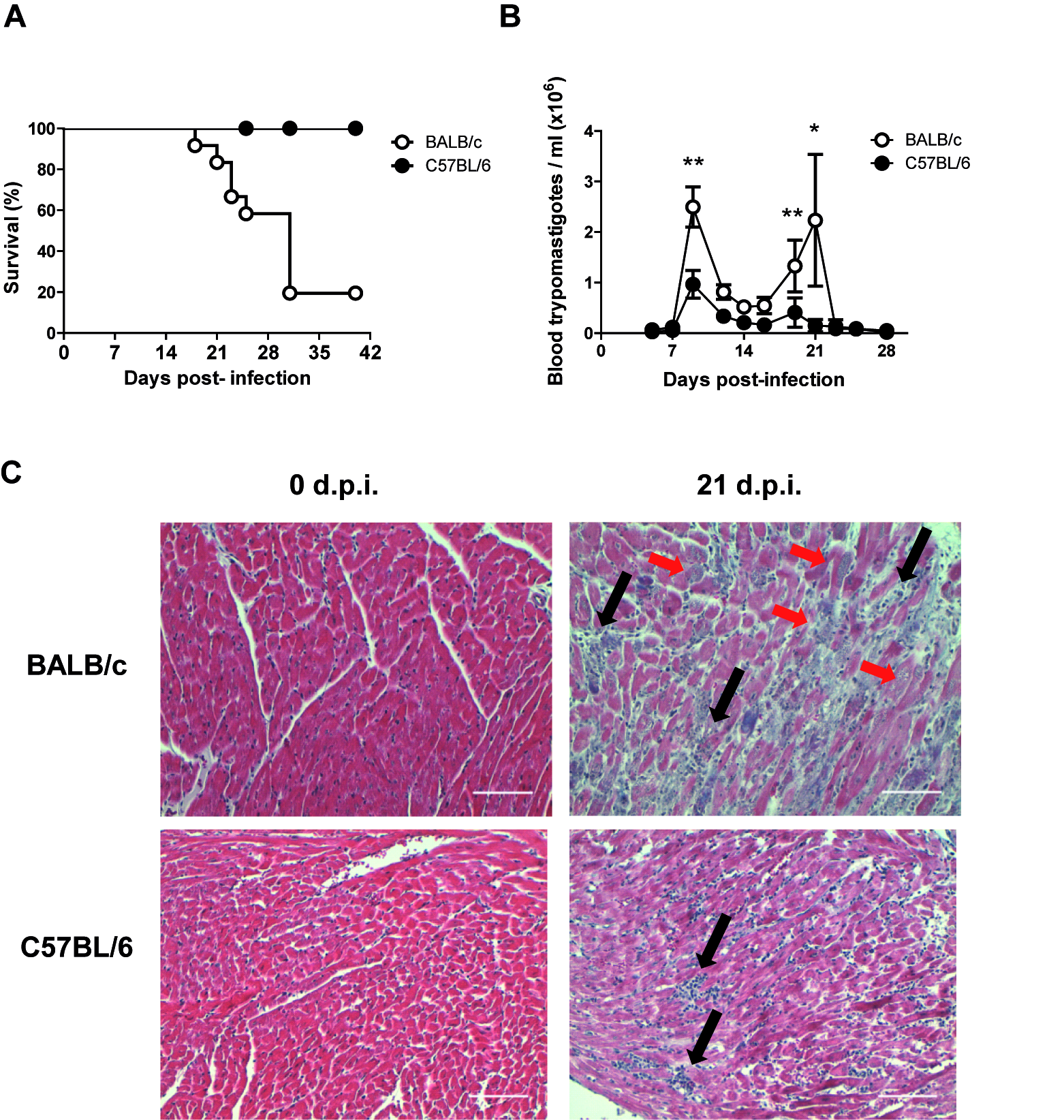

Supplement: S1 Fig — (A) Survival was checked every day during the infection in BALB/c and C57BL/6 mice (n = 5). (B) The presence of the parasites in the blood of BALB/c and C57BL/6 mice was quantified by direct counting under optical microscopy. Means ± SEM of a representative experiment (n = 4) from two independent experiments are shown. (* p< 0.05; ** p< 0.01). (C) Histology of cardiac tissue in non-infected (0 d.p.i.) or 21 d.p.i T. cruzi infected BALB/c and C57BL/6 mice. Representative pictures of heart tissue sections of each group, processed for Masson`s Trichrome histology staining, are shown. Black arrows point to the infiltrating leukocytes and red arrows point to parasite nests. Scale bar is 100 μm. (TIF) [file pntd.0004025.s001.tif]

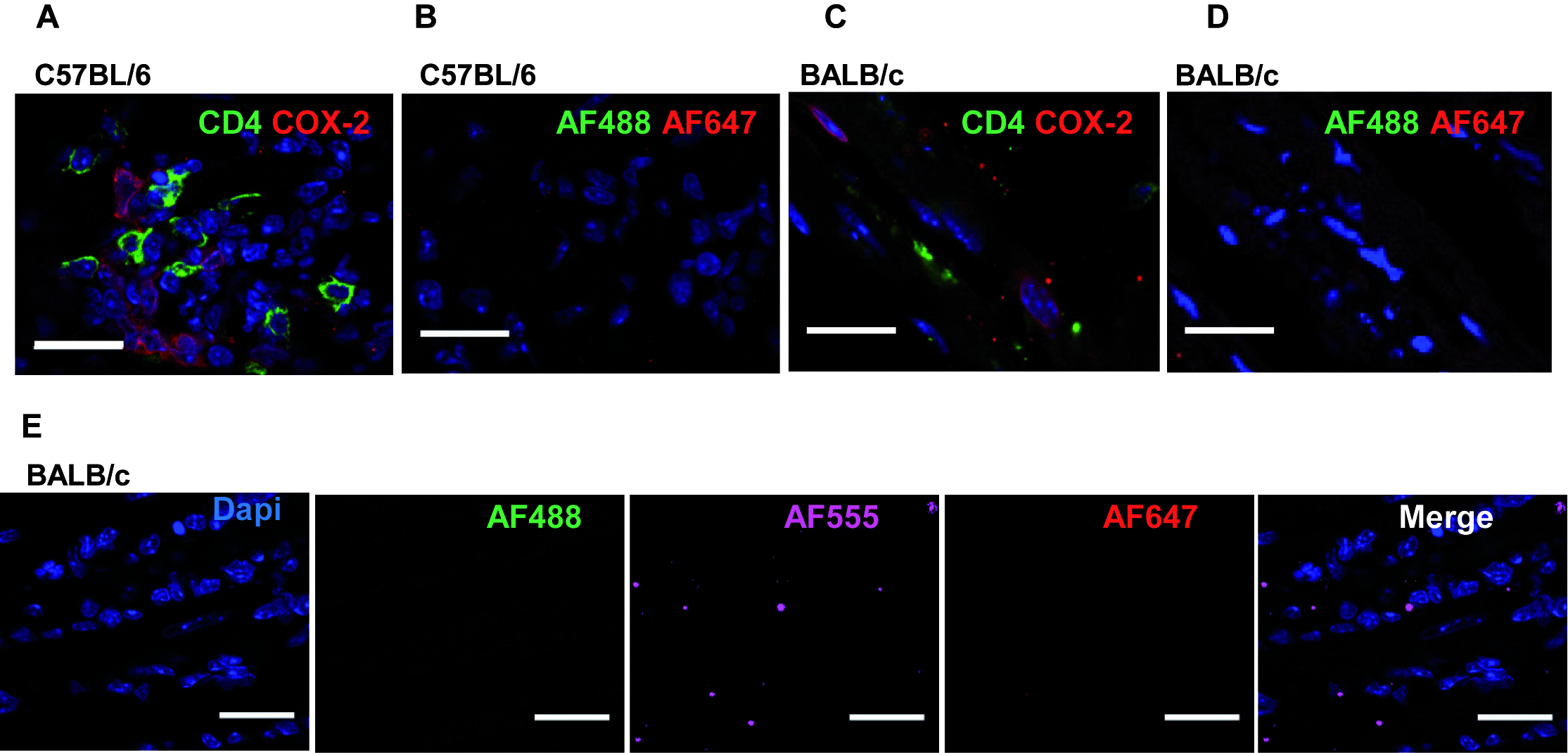

Supplement: S2 Fig — (A) Heart tissue was isolated at 14 d.p.i. from infected C57BL/6 mice and sections were stained with DAPI for nuclei (Blue), the lymphocyte marker CD4 (green) or COX-2 (red). A representative picture of several sections analyzed in at least three different mice from two independent experiments is shown. (B) Control sample from heart of C57BL/6 mice at 14 d.p.i. incubated with secondary antibodies coupled to Alexa Fluor (AF) 488, and 647 in the absence of primary antibodies. (C) Same as in A from heart of BALB/c mice at 21 d.p.i.. (D) Same as in B from heart of BALB/c mice at 21 d.p.i. (E) Control samples from heart of BALB/c mice at 21 d.p.i. incubated with secondary antibodies coupled to Alexa Fluor (AF) 488, 555 and 647 in the absence of primary antibodies. Scale bar is 20 μm. (TIF) [file pntd.0004025.s002.tif]

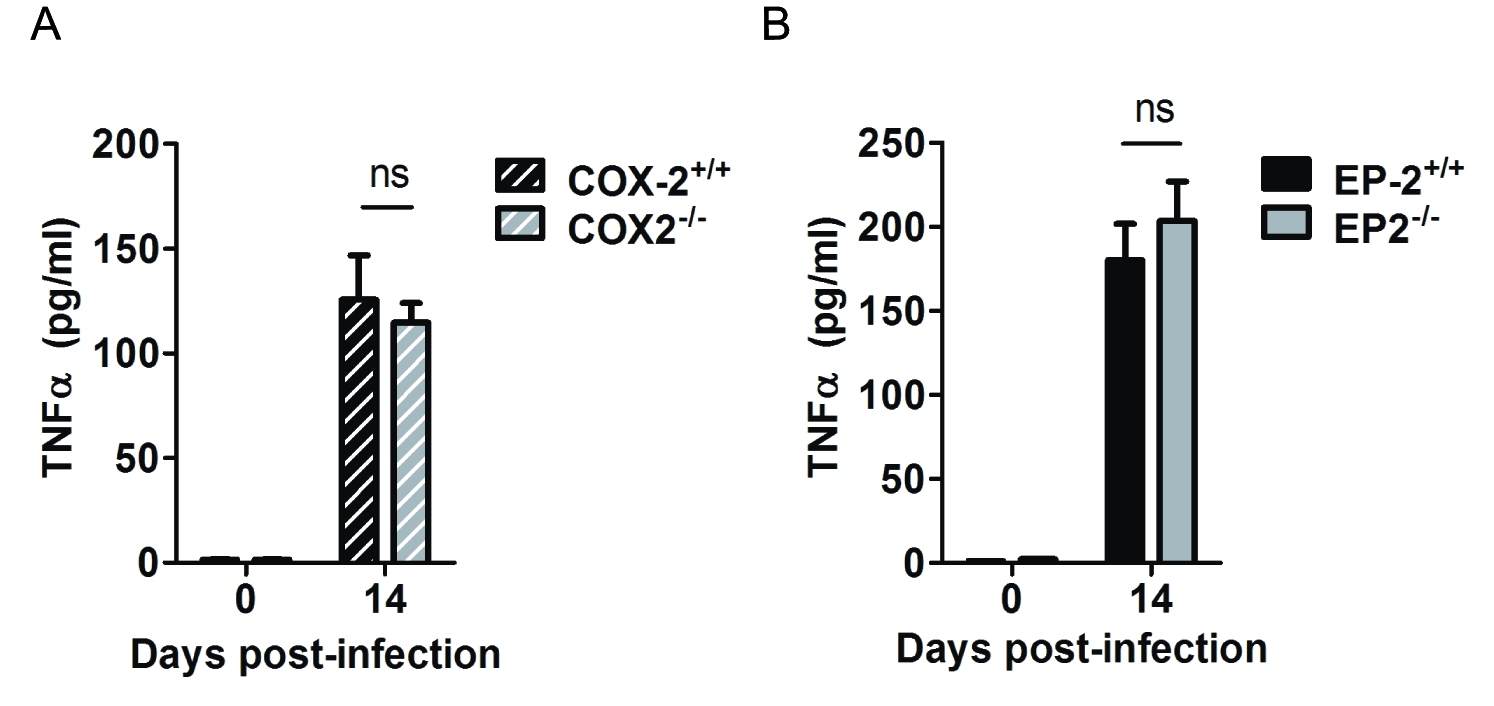

Supplement: S3 Fig — TNFα concentration in non-infected mice (0 d.p.i.) and at 14 d.p.i. in blood serum of (A) COX-2+/+ and COX-2-/- and (B) EP-2+/+ and EP-2-/- mice. Representative means ± SEM from two independent experiments are shown (n = 6) (ns = non-significant). (TIF) [file pntd.0004025.s003.tif]

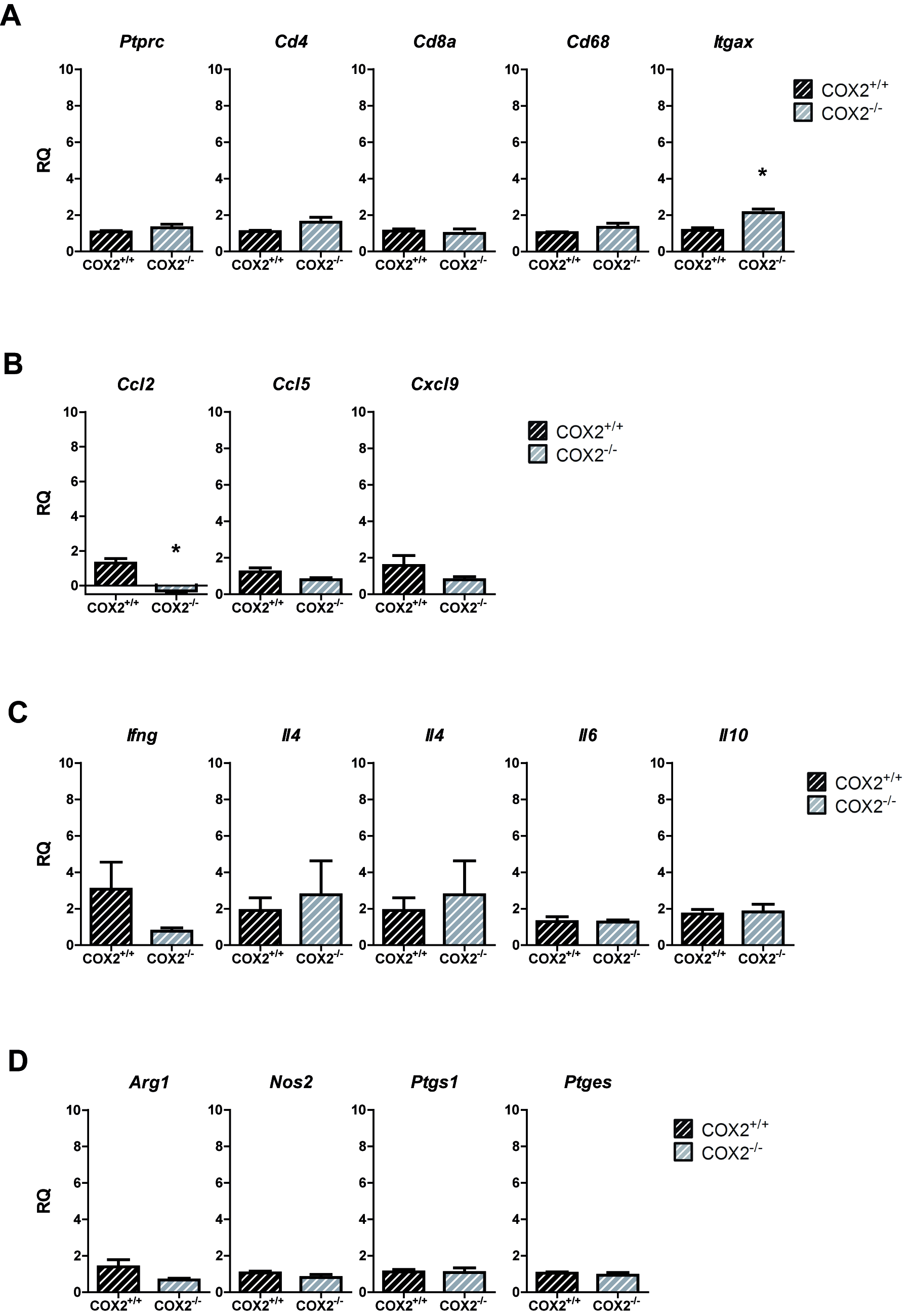

Supplement: S4 Fig — mRNA levels of the different genes analyzed was determined by qRT-PCR in heart tissue RNA samples isolated from non-infected (0 d.p.i.) COX-2+/+ or COX-2-/- mice. Data are expressed as RQ calculated from CT values as described in Methods. Gene expression of lymphoid and myeloid cell markers as Ptprc, Cd4, Cd8a, Cd68 and Itgax (A), chemokines as Ccl2, Ccl5 and Cxcl9 (B), cytokines as Ifng, Tnf, Il4, Il6 and Il10 (C) and enzymes as Arg1, Nos2, Ptgs1 and Ptges (mPGES1) (D) is shown. Means ± SEM from one representative experiment (n = 3) out of four is shown (n = 5; * p<0.05). (TIF) [file pntd.0004025.s004.tif]

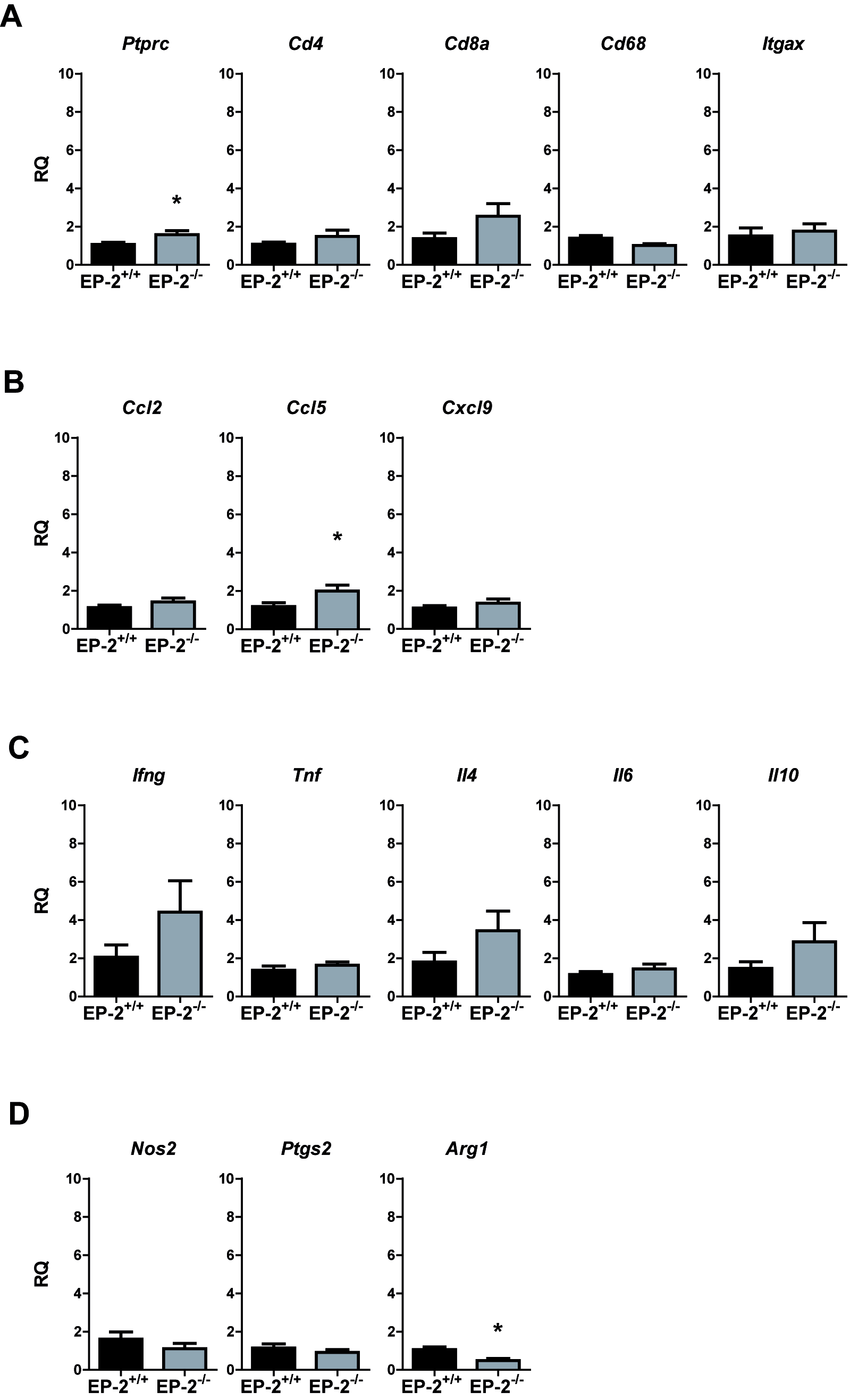

Supplement: S5 Fig — mRNA levels of the different genes analyzed was determined by qRT-PCR in heart tissue RNA samples isolated from non-infected (0 d.p.i.) EP-2+/+ or EP-2-/- mice. Data are expressed as RQ calculated from CT values as described in Methods. Gene expression of lymphoid and myeloid cell markers as Ptprc, Cd4, Cd8a, Cd68 and Itgax (A), chemokines as Ccl2, Ccl5 and Cxcl9 (B), cytokines as Ifng, Tnf, Il4, Il6 and Il10 (C) and enzymes as Nos2, Ptgs2 and Arg1 (D) is shown. Means ± SEM from one representative experiment (n = 3) out of four is shown (n = 5; * p<0.05). (TIF) [file pntd.0004025.s005.tif]
