# Supplementary material for: Cyclooxygenase-2 and Prostaglandin E2 Signaling through Prostaglandin Receptor EP-2 Favor the Development of Myocarditis during Acute Trypanosoma cruzi Infection
Source: PLoS Negl Trop Dis. 2015 Aug 25;9(8):e0004025. doi: 10.1371/journal.pntd.0004025 (PMC4549243; doi:10.1371/journal.pntd.0004025)
Supplement: S1 Table — List of the Taqman Probes from Applied Biosystems (A&B) used in the mRNA analysis by quantitative RT-PCR, including reference of the manufacturer, gene symbol and protein name. (PDF) [file pntd.0004025.s006.pdf]

S1 Table. Taqman probes. List of the Taqman Probes from Applied Biosystems (A&B) used in the mRNA analysis by quantitative RT-PCR, including reference of the manufacturer, gene symbol and protein name.

CODE (A&B)	GENE	PROTEIN
Mm 00477214_m1	*Ptgs1*	Cyclooxygenase 1, COX-1
Mm 00478374_m1	*Ptgs2*	Cyclooxygenase 2, COX-2
Mm 00452105_m1	*Ptges*	Prostaglandin E synthase, mPGES1
Mm01330613_m1	*Ptgds*	Lipocalin type prostaglandin D synthase, LPGDS
Mm 00479846_m1	*Hpgds*	Hematopoietic prostaglandin D synthase, HPGDS
Mm00495553_m1	*Tbxas1*	Thromboxane synthase, TXAS
Mm 99999056_m1	*Ccl2*	Chemokine (C-C motif) ligand 2, MCP-1
Mm 01302427_m1	*Ccl5*	Chemokine (C-C motif) ligand 5, RANTES
Mm 00434946_m1	*Cxcl9*	Chemokine (C-X-C motif) ligand 9, MIG
Mm 00443258_m1	*Tnf*	Tumor necrosis factor alpha, TNF-α
Mm 00801778_m1	*Ifng*	Gamma interferon , IFN-γ
Mm 00446190_m1	*Il6*	Interleukin 6, IL-6
Mm 00439616_m1	*Il10*	Interleukin 10, IL-10
Mm 00445259_m1	*Il4*	Interleukin 4, IL-4
Mm 00442754_m1	*Cd4*	CD4 antigen, CD4
Mm 00839636_g1	*Cd68*	CD68 antigen, CD68
Mm 01182107_g1	*Cd8a*	CD8 antigen, alpha chain, CD8
Mm 00498698_m1	*Itgax*	Integrin alpha X, CD11c
Mm 00802530_m1	*Emr1*	F4/80
Mm00448463_m1	*Ptprc*	CD45
Mm 00475988_m1	*Arg1*	Arginase-1, Arg-1
Mm 00440502_m1	*Nos2*	Nitric oxide synthase 2, iNOS

Mm 00443097_m1	*Ptger1*	EP-1 receptor
Mm 00436051_m1	*Ptger2*	EP-2 receptor
Mm 00441045_m1	*Ptger3*	EP-3 receptor
Mm 00436053_m1	*Ptger4*	EP-4 receptor
Hs99999901_s1	*18S*	Eukaryotic 18S rRNA
