# Supplementary material for: Cyclooxygenase-2 and Prostaglandin E2 Signaling through Prostaglandin Receptor EP-2 Favor the Development of Myocarditis during Acute Trypanosoma cruzi Infection
Source: PLoS Negl Trop Dis. 2015 Aug 25;9(8):e0004025. doi: 10.1371/journal.pntd.0004025 (PMC4549243; doi:10.1371/journal.pntd.0004025)
Supplement: S2 Table — List of antibodies from different providers used in confocal immunofluorescence and western blot analysis including the reference from each provider, the application and the dilution utilized. (PDF) [file pntd.0004025.s007.pdf]

S2 Table. Antibodies. List of antibodies from different providers used in confocal immunofluorescence and western blot analysis including the reference from each provider, the application and the dilution utilized.

ANTIBODY	REFERENCE	PROVIDER	APPLICATION	DILUTION
Rabbit anti-iNOS	sc-650	Santa Cruz Biotechnology	WB,IHF	1:1000, 1:200
Rabbit anti-COX-2	160126	Cayman Chemical	WB	1:500
Rabbit anti-COX-2	160106	Cayman Chemical	IHF	1:200
Goat anti-Actin	sc-1616	Santa Cruz Biotechnology	WB	1:1000
Goat anti-Arg-1	sc-18354	Santa Cruz Biotechnology	WB, IHF	1:1000, 1:200
Rat anti-CD68	MCA1957GA	AbD Serotec	IHF	1:200
Rat anti-CD4	557681	BD Pharmigen	IHF	1:200
Donkey anti-Rat IgG AF-488	A-21208	Invitrogen	IHF	1:200
Donkey anti-Rabbit IgG AF-647	A-31573	Invitrogen	IHF	1:200
Rabbit anti-Goat IgG AF-555	A-21431	Invitrogen	IHF	1:200
