# Supplementary material for: Cyclooxygenase-2 and Prostaglandin E2 Signaling through Prostaglandin Receptor EP-2 Favor the Development of Myocarditis during Acute Trypanosoma cruzi Infection
Source: PLoS Negl Trop Dis. 2015 Aug 25;9(8):e0004025. doi: 10.1371/journal.pntd.0004025 (PMC4549243; doi:10.1371/journal.pntd.0004025)
Supplement: S1 File — Plugin Trainable Weka Segmentation developed by Ignacio Arganda Carreras (Versailles, France). (PDF) [file pntd.0004025.s008.pdf]

S1 file. Image J macro used for automated image analysis. Plugin Trainable Weka Segmentation developed by Ignacio Arganda Carreras (Versailles, France).

```
origen = getDirectory(“Elige la carpeta con las imagenes originales a procesar”);

resultados = getDirectory(“Elige la carpeta para guardar los resultados “);

lista = getFileList(origen);

setBatchMode(false);

for (z=0; z<lista.length; z++) {

showProgress(z+1, lista.length);

open(origen+lista[z]);

nombre = lista[z];

run(“Advanced Weka Segmentation”, “origen + nombre”);

selectWindow(“Advanced Weka Segmentation”);

call(“trainableSegmentation.Weka_Segmentation.loadClassifier”, “C:\\Users\\vlabrador\\Desktop\\clasificador.model”);

wait(3000);

call(“trainableSegmentation.Weka_Segmentation.applyClassifier”, origen, nombre, “showResults=true”, “storeResults=false”, “probabilityMaps=false”, “”);

selectWindow(“Advanced Weka Segmentation”);

selectWindow(“Classification result”);

saveAs(“.tiff”, resultados + nombre + “-Binary”);

run(“Set Scale...”, “distance=313.67 known=100 pixel=1 unit=µm global”);

run(“Set Measurements...”, “area area_fraction limit display redirect=None decimal=3”);

class1 = nombre + “__Fibrosis”;

rename(class1);

//run(“Threshold...”);

setThreshold(175, 255);

run(“Measure”);
```

```
class2 = nombre + “__Fondo”;
rename(class2);
//run(“Threshold...”);
setThreshold(150, 174);
run(“Measure”);
class3 = nombre + “__Nucleos”;
rename(class3);
//run(“Threshold...”);
setThreshold(75, 100);
run(“Measure”);
class4 = nombre + “__Tejido y Eritrocitos”;
rename(class4);
//run(“Threshold...”);
setThreshold(0, 74);
run(“Measure”);
close();
close();
close();
close();
}
selectWindow(“Results”);
saveAs(“Text”, resultados + “Resultados.xls”);
run(“Close”);
```
